# Supplementary material for: Highly efficient gene knockout in tumor-infiltrating lymphocytes by adenine base editing
Source: Mol Ther Oncol. 2025 Aug 22;33(3):201041. doi: 10.1016/j.omton.2025.201041 (PMC12696495; doi:10.1016/j.omton.2025.201041)
Supplement: Document S1. Figures S1–S13 and Tables S1 and S2 [file mmc1.pdf]

OMTON, Volume *33*

Supplemental information

Highly efficient gene knockout in tumor-infiltrating lymphocytes by adenine base editing

Morteza Hafezi, Raphael Genolet, Leila Hadadi, Bovannak Stewen Chap, Sara Bobisse, Greta Maria Paola Giordano Attianese, Hanan El Jorfi, Daniela Cropp, Laetitia Pericou, Marion Arnaud, Kirsten Scholten, Typhanie Maurouard, Denarda Dangaj Laniti, Alexandre Harari, Bernhard Gentner, Melita Irving, and George Coukos

Tabel S1: ABE matched guide RNA sequences.

Target genes	Guide RNA sequence (no PAM)
TIM3 guide RNA1	5'-CTTACTGTTAGATTTATATC-3'
TIM3 guide RNA2	5'-GTTACCTGGGCCATGTCCCC-3'
TIGIT guide RNA1	5'-CAGGAATACCTGAGCTTTCT-3'
TIGIT guide RNA2	5'-CAGGCCTTACCTGAGGCGAG-3'

Table S2: Primers used for sanger sequencing and NGS analysis.

Nextera adapter-TIM3 forward	5'-TCGTCGGCAGCGTCAGATGTGTATAAGAGACAGAAGTTGATTCCTGGGTGTTCAG-3'
Nextera adapter-TIM3 reverse	5'-GTCTCGTGGGCTCGGAGATGTGTATAAGAGACAGGATTTCCCCTCCAAGTTGAGTA-3'
Nextera adapter-TIGIT forward	5'-TCGTCGGCAGCGTCAGATGTGTATAAGAGACAGCTACCCTGATGGGACGTACACT-3'
Nextera adapter-TIGIT reverse	5'-GTCTCGTGGGCTCGGAGATGTGTATAAGAGACAGCACCTGGAAATGCTTTTAGACC-3'
Nextera adapter forward	5'-TCGTCGGCAGCGTCAGATGTGTATAAGAGACAG-3'
Nextera adapter reverse	5'-GTCTCGTGGGCTCGGAGATGTGTATAAGAGACAG-3'

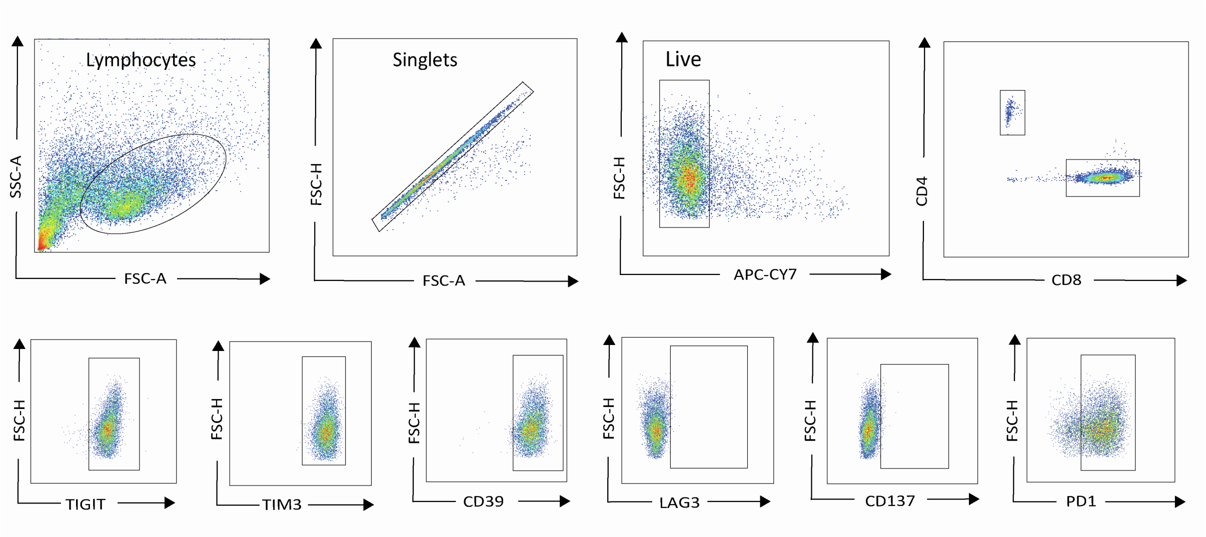

Figure S1: Gating strategy for immunophenotyping of TILs. Exemplary plot for exhaustion and activation markers in live CD4^{+} and CD8^{+} TILs. Doublet cells were excluded from the gated TIL population using FSC-A/FSC-H parameters.

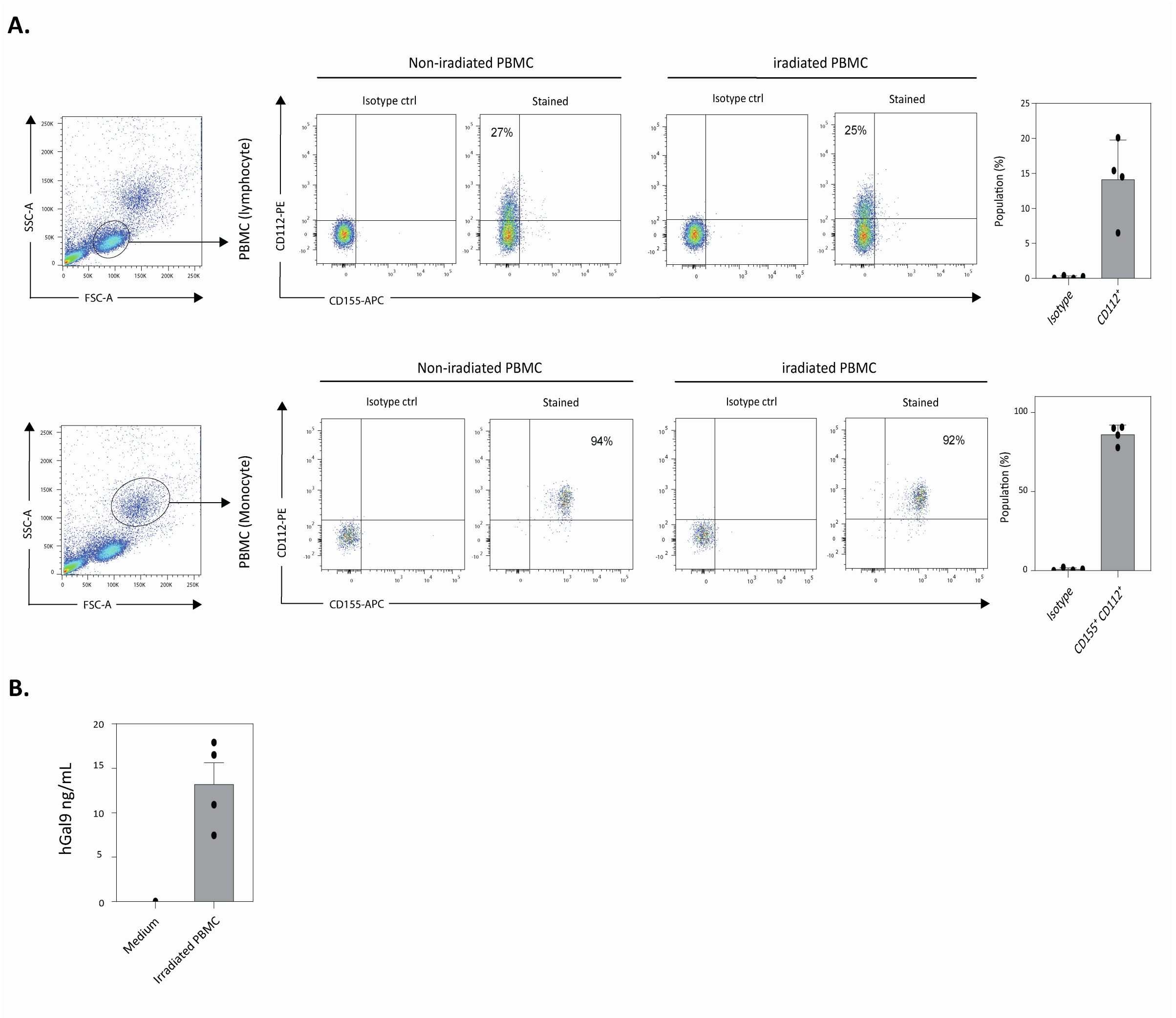

Figure S2: Expression of TIGIT- and TIM3-specific ligands in irradiated feeder cells. (A) Flow cytometry plots demonstrate the expression of TIGIT specific ligands CD112 and CD155 in non-irradiated and irradiated PBMC (**top:** lymphocyte; **bottom:** monocyte). Bar plots represent pooled data from n=4 biological replicates. Isotype control-stained TILs were used as a negative-control for the experiment. Bar plot represents pooled data from n=4 different melanoma-specific TILs (**top:** lymphocyte; **bottom:** monocyte). Lymphocytic and monocytic populations were defined based on their FSC-A and SSC-A parameters. Isotype-stained TILs were used as controls. **(B)** Bar plots represent soluble hGAL9 of irradiated PBMC at day 4 post activation (REP medium). Data pooled data from n=4 biological replicates. REP medium alone was used as negative control.

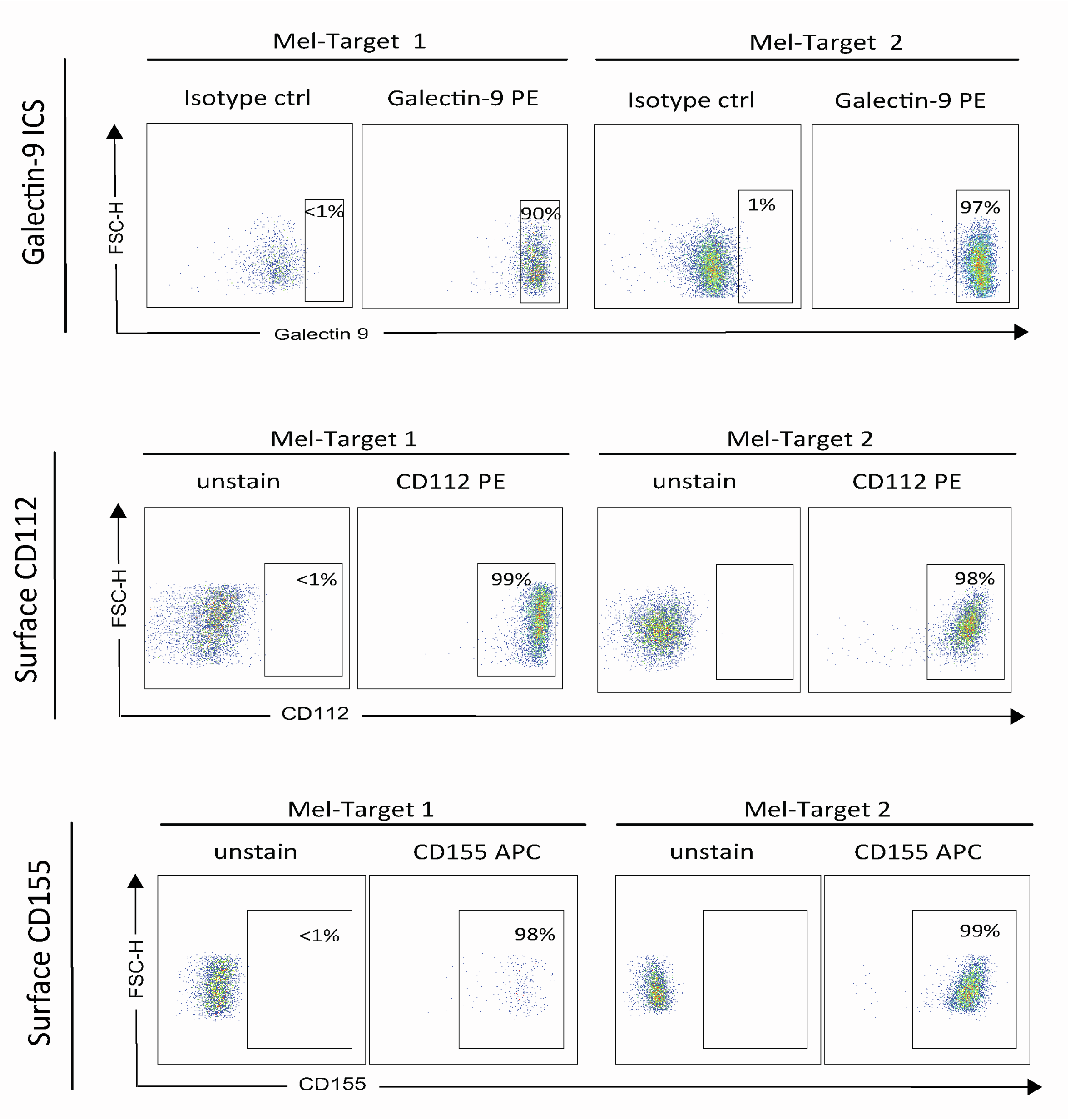

Figure S3: Expression of TIM-3- and TIGIT-specific ligands in patient-derived melanoma cell lines. Exemplary dot plots show the expression of **top**: intracellular staining (ICS) of Galectin-9, **middle**: surface expression of CD112, and **bottom**: surface expression of CD155 in melanoma tumor cell lines.

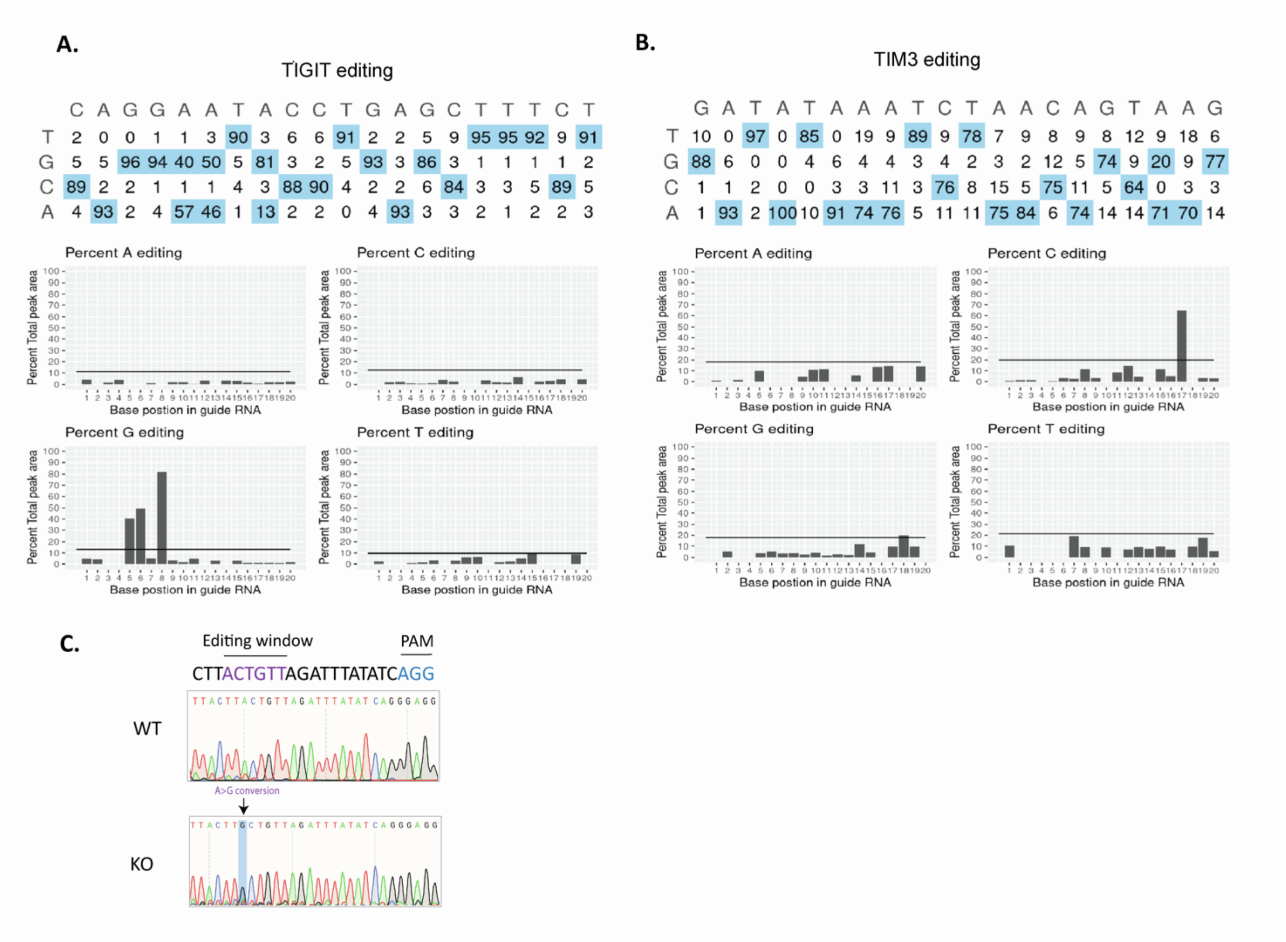

Figure S4: Validation of targeted mutations in ABE-TILs. (**A, B**). **Top:** editing table plot demonstrates the proportion of base conversions within the editing window. **Bottom:** editing bar plots demonstrate the percent of As (antisense Ts) are converted to Gs (antisense Cs). All graphs and plots were generated by analyzing a sanger sequencing file using EditR (n=1). (**C**) Chromatograms obtained by Sanger sequencing demonstrate the point mutation introduced by ABE in exemplary TIM3 edited TILs.

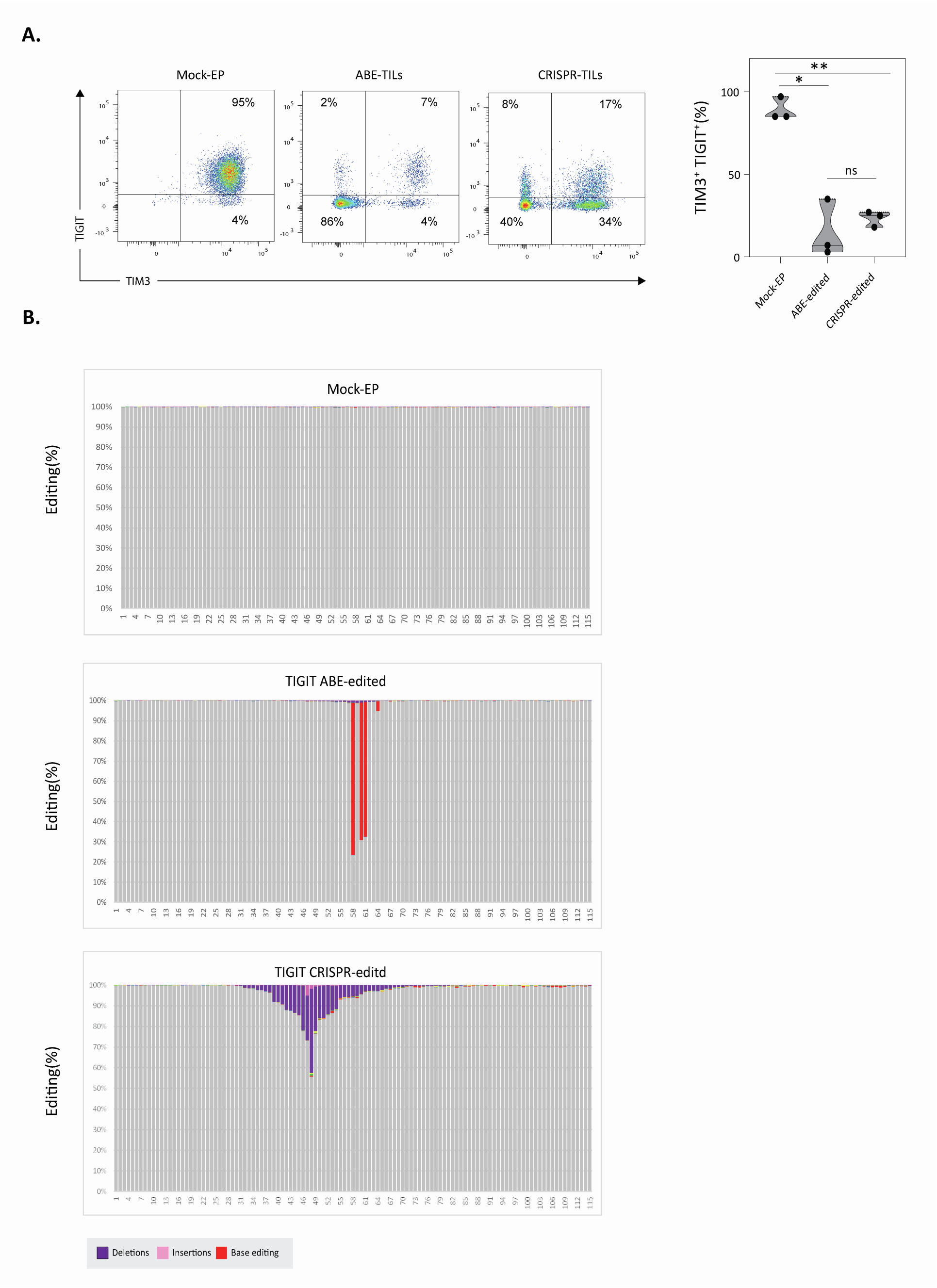

Figure S5: Indel characterization post ABE and CRISPR-CAS9 editing of TILs. (A) Left: Flow cytometry plots demonstrating TIGIT and TIM3 KO in TILs by ABE and CRISPR-Cas9 editing. **Right:** Plot demonstrating % dual KO of TIGIT and TIM3 in ABE versus CRISPR-Cas9 edited TILs (n=3 different melanoma specific TIL samples). **(B)** Exemplary plots showing indel events as evaluated by deep sequencing post ABE (**middle**) or CRISPR-Cas9 (**bottom**) editing in the TIGIT locus. Plot is exemplary of n=3 different melanoma-specific TILs (purple: deletion events; pink: insertion events; red: base editing events). Statistical analysis was performed by one-way ANOVA with Tukey's multiple comparison test. Statistical significance indicated as *P < 0.05, **P < 0.01. ns, not significant.

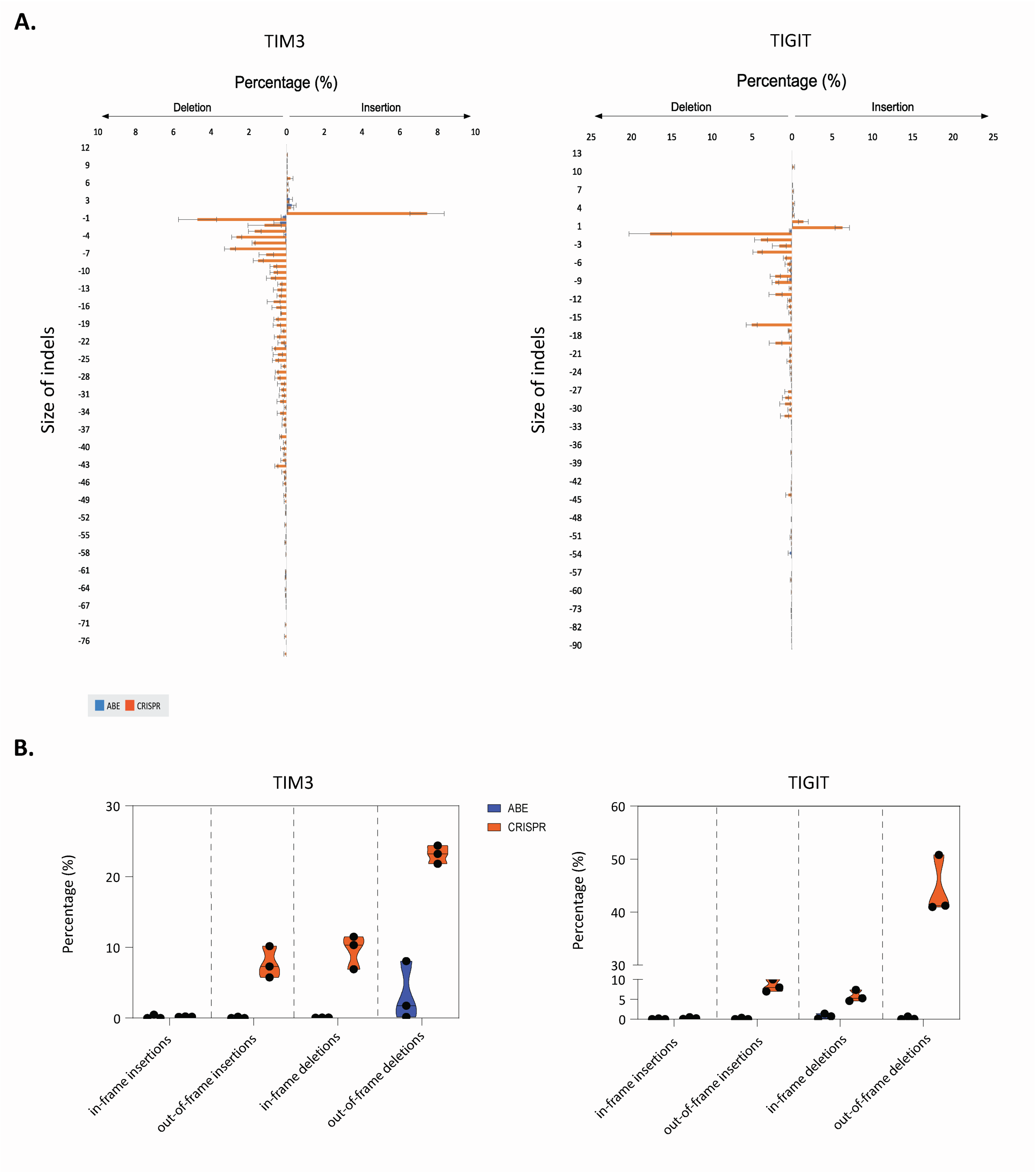

Figure S6: Analysis of ABE versus CRISPR-Cas9 edited TILs. (A) Plot demonstrating the frequency and size of indels for both TIM3 (left) and TIGIT (right) target locus in both CRISPR and ABE edited TILs analyzed by NGS (n=3 biological replicates). **(B)** Violin plots showing percentage of transcripts with in-frame insertion, out-frame insertion, in-frame deletion and out-frame deletion in in both CRISPR and ABE edited TILs. For (B), all values are subtracted from background observed in mock-electroporated (i.e., WT genome) TILs.

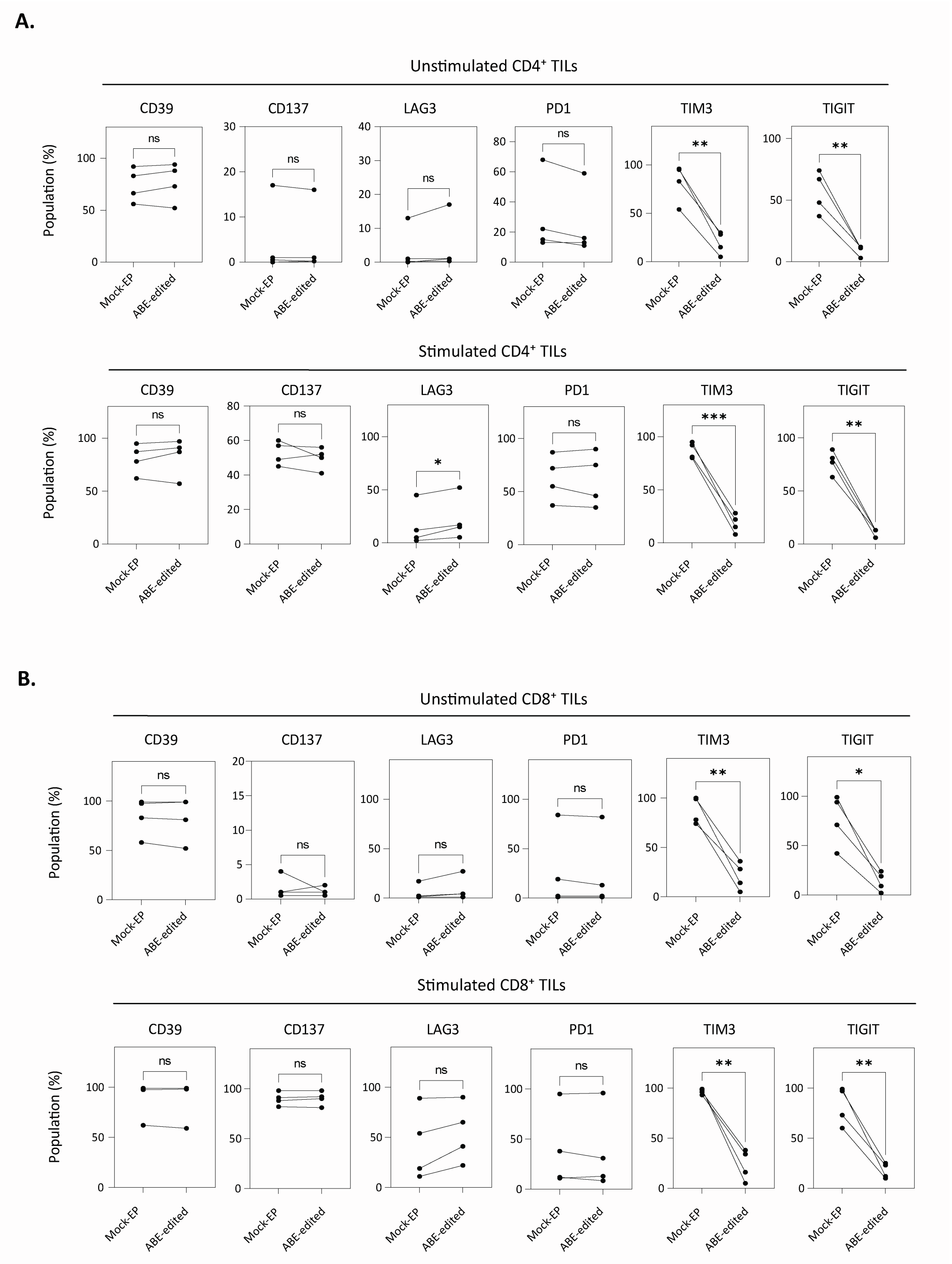

Figure S7: Phenotype of adenine based edited versus non-edited TILs. (A-B) Bar plots demonstrating activation/exhaustion markers in resting (top) and stimulated (bottom) **(A)** $CD4^+$ and **(B)** $CD8^+$ subset of Ova- and Mel-TILs. For Statistical analysis paired t test was performed. Statistical significance indicated as *$P < 0.05$, **$P < 0.01$ and ***$P < 0.001$. ns; not significant.

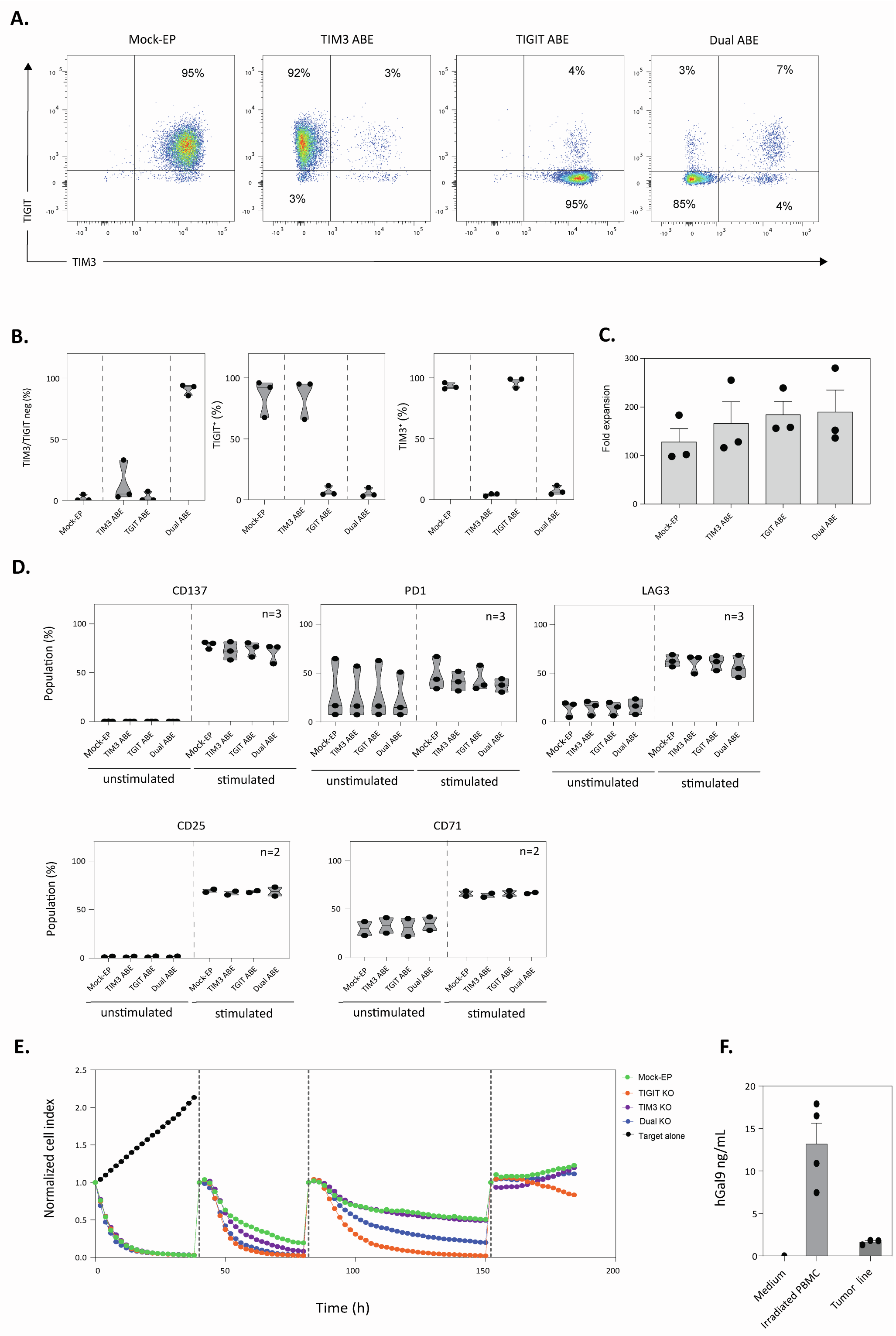

Figure S8: Comparison of single and dual TIM3 and TIGIT ABE-edited TILs (A) Exemplary dot plot showing single and dual KO efficiency in ABE-edited TILs. **(B)** Violin plots demonstrating editing efficiency (n=3 biological replicates). **Left:** percentage of double negative TIM3/TIGIT TILs, **middle:** percentage of TIGIT^{+} TILs, **right:** percentage of TIM3^{+} TILs. **(C)** Fold-expansion calculated at the end of REP phase for all edited and non-edited

TILs (n=3 biological replicates). **(D)** Immunophenotyping of single and dual ABE-edited TILs. Violin plots demonstrate activation/exhaustion markers in unstimulated and stimulated TILs. The number of biological replicates is shown in each violin plot. **(E)** Exemplary plot showing serial cytolytic capacity of TILs determined by a real-time killing assay. The plot is exemplary of 3 independent experiments. Edited and non-edited TILs were co-cultured at an effector-to-target ratio of 2:1 with autologous melanoma target cells. T cell-mediated cytotoxicity was determined by measuring GFP-positive tumor cells at 2-hour intervals for approximately 90 hours post T cell addition using the Incucyte S3 system. **(F)** Bar plots represent soluble hGAL9 levels for both irradiated PBMCs at day 4 post-activation and the patient-derived melanoma line. Data pooled data from n=4 biological replicates for PBMC and n=3 technical replicates of patient-derived tumor line. REP medium alone is used as a negative control.

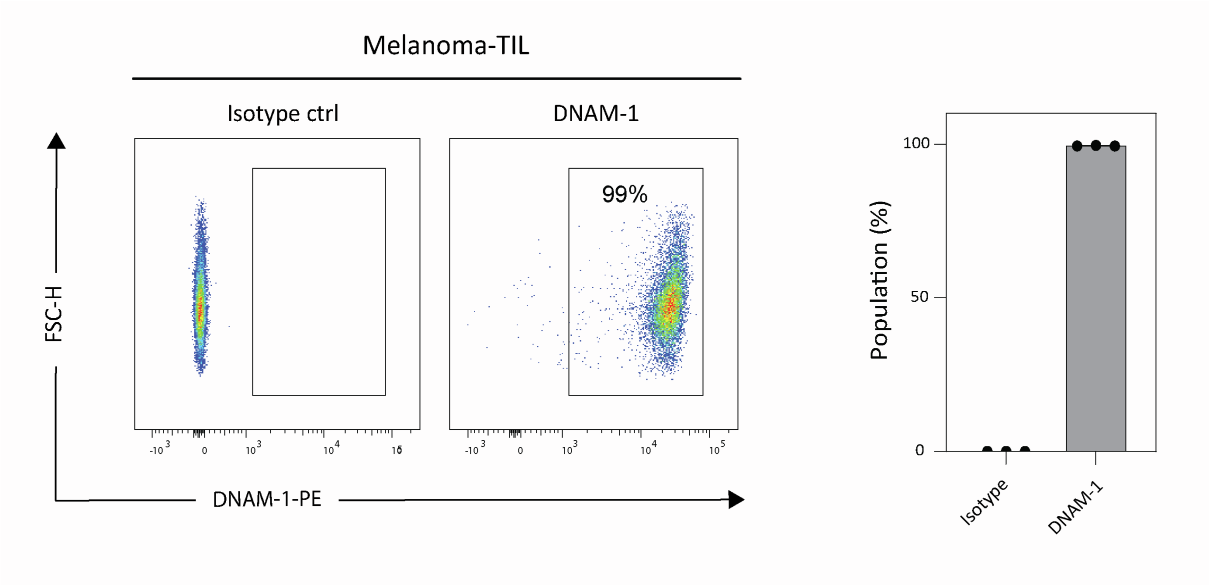

Figure S9: Evaluation of DNAM-1 expression on REP-TILs. Exemplary flow cytometry plots show the expression of DNAM-1 melanoma TILs. Bar plots represent pooled data from n=3 different melanoma-specific TILs. Isotype control-stained (Isotype ctrl) TILs were used as negative-control for the experiment.

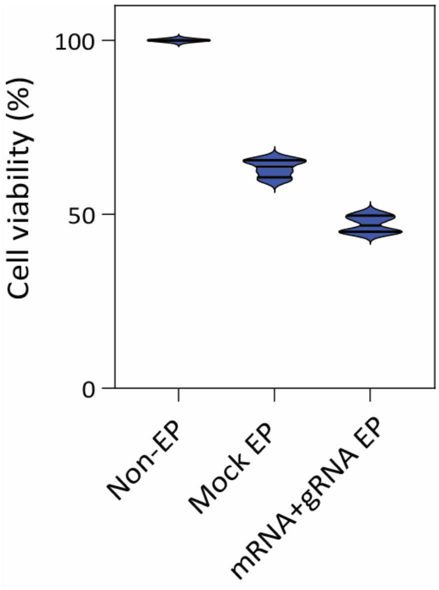

Figure S10: Viability of TILs post-electroporation. Bar plots represent pooled data from 4 different TIL samples. Electroporated (EP) and non-EP TILs were counted after EP using Trypan blue. Non-EP TIL counts were set to 100%.

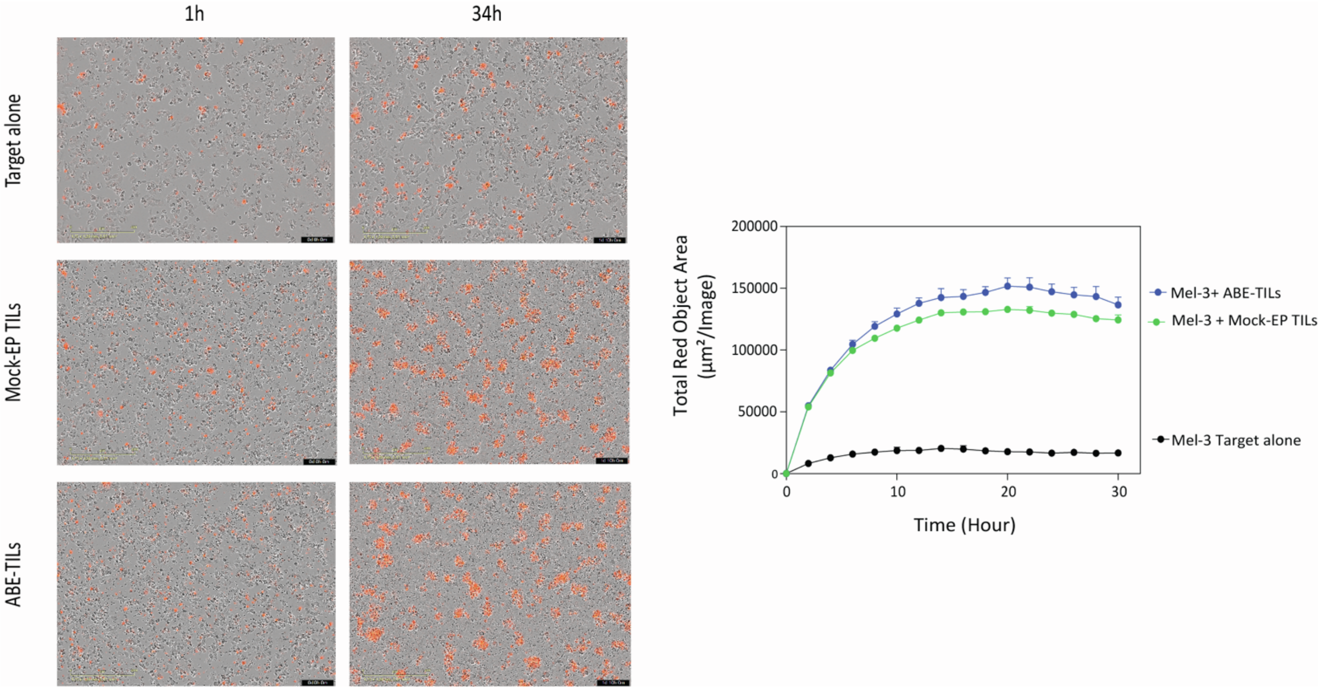

Figure S11: Dual TIM3 and TIGIT ABE-TILs retain unaltered killing capacity in the initial tumor cell challenge. Left: Exemplary images show the killing efficiency of Mock and ABE-EP TILs after 35 hours co-culture. ABE- and Mock-EP TILs were co-cultured at an effector: target ratio of 1:1 with autologous melanoma target cells (Mel-3 target cell line). **Right:** Plot illustrates T cell-mediated cytotoxicity evaluated using the IncuCyte S3 system by measuring Cytox red positive cells at 2-hour intervals for approximately 35 hours post co-culture initiation. Melanoma target cells alone were used as a negative control.

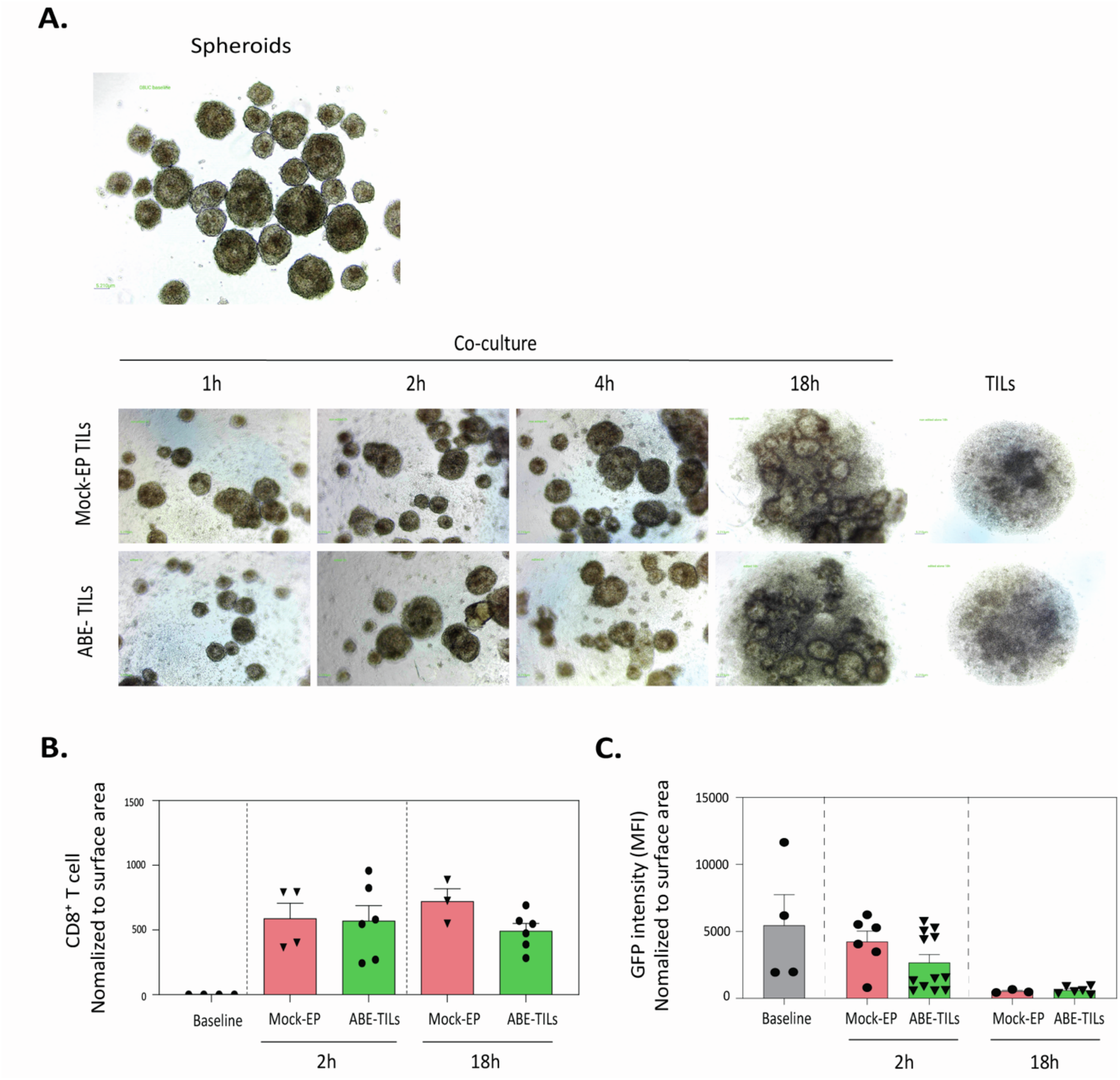

Figure S12: Characterization of melanoma spheroid-TIL co-cultures. (A) Exemplary brightfield microscopy images of spheroids alone and co-cultured with Mock-EP and ABE-TILs at different time-points. Images were acquired using the 5x objective. **(B)** evaluation of T cell infiltration in tumor spheroids at 2 and 18 hours post-co-culture. **(C)** GFP fluorescence intensity within the region of interest (ROI) in the spheroid at baseline, and after 2 and 18 hours co-culture.

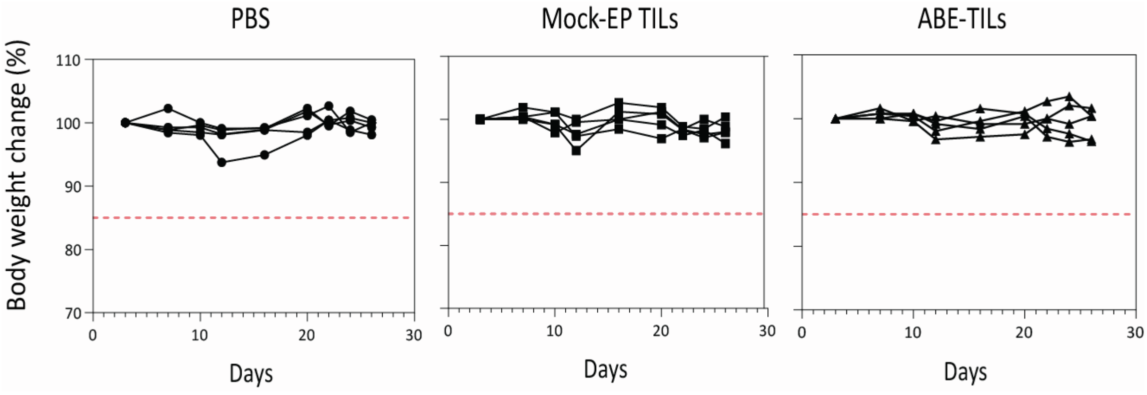

Figure S13: Weight measurements of NSG mice following TIL administration. Graphs illustrate body weight changes for mice over time following control PBS injections versus Mock-EP + IL-2 or ABE-TIL + IL-2 treatments. Each line represents the percentage of body weight changes in a single mouse. Initial body weight was set as 100% and the red dashed line marks 15% weight loss at which point mice would be sacrificed according to regulations.
