# Supplementary material for: Highly efficient gene knockout in tumor-infiltrating lymphocytes by adenine base editing
Source: Mol Ther Oncol. 2025 Aug 22;33(3):201041. doi: 10.1016/j.omton.2025.201041 (PMC12696495; doi:10.1016/j.omton.2025.201041)
Supplement: Document S2. Article plus supplemental information [file mmc2.pdf]

Molecular Therapy
Oncology
Original Article

Highly efficient gene knockout in tumor-infiltrating lymphocytes by adenine base editing

Morteza Hafezi,[1] Raphael Genolet,[1] Leila Hadadi,[1] Bovannak Stewen Chap,[1] Sara Bobisse,[1] Greta Maria Paola Giordano Attianese,[1] Hanan El Jorfi,[1] Daniela Cropp,[1] Laetitia Pericou,[1] Marion Arnaud,[1] Kirsten Scholten,[1] Typhanie Maurouard,[2] Denarda Dangaj Laniti,[1] Alexandre Harari,[1] Bernhard Gentner,[1] Melita Irving,[1] and George Coukos[1]

[1]Ludwig Institute for Cancer Research, Department of Oncology, University of Lausanne and Lausanne University Hospital (CHUV), Lausanne, Switzerland; [2]MaxCyte Inc., Rockville, MD 20850, USA

The disruption of immune checkpoints in T cells is a promising tool for improving the efficacy of tumor-infiltrating lymphocyte (TIL) therapy. While CRISPR-Cas9 genome-editing is efficient, Cas9 nucleases induce double-strand DNA breaks and risks improper translocations, inversions, and chromosomal deletions in engineered T cells. Cas9 nickase (nCas9) used in base-editing cuts only a single strand of DNA, reducing genetic aberrations in modified cells. Here, we established a small-scale, good manufacturing practice-compatible adenine base editing (ABE) procedure for both single and dual knockout of co-inhibitory receptors TIM3 and TIGIT in TILs. ABE-mediated conversion of A·T to G·C pairs in TIM3 and TIGIT specific splice-sites led to high knockout efficiency, with negligible insertion-deletion events post editing. Using melanoma and ovarian TILs, we show that target-specific editing by ABE of TIM3 and TIGIT improved (1) TIL fold-expansion during the rapid expansion protocol without adversely impacting phenotype, (2) cytokine production, and (3) serial killing upon co-culture with autologous patient-derived tumor cells *in vitro*. Moreover, dual edited TILs were able to infiltrate tumor spheroids *in vitro* and control patient-derived tumors *in vivo*. Taken together, we show the feasibility of ABE multiplex editing as a promising tool for engineering TILs for clinical applications.

INTRODUCTION

The adoptive cell transfer (ACT) of autologous TILs (TIL-ACT) is a personalized cancer therapy based on the infusion of polyclonal T cells expanded *ex vivo* from surgically removed autologous tumors.[1,2] TIL-ACT has been extensively tested in metastatic melanoma, an immunogenic cancer,[2–4] and the encouraging clinical outcomes of this therapy (e.g., NCT02360579) led to the US Food and Drug Administration (FDA) approval of Lifileucel in February 2024, the first cellular therapy for a solid cancer.[5] Notably, objective responses have also been induced by TIL-ACT in patients with metastatic epithelial cancers such as head and neck, breast, ovarian and colon, upon use of methods to selectively expand tumor-antigen specific T cells.[6–8]

Despite clinical gains to date, various factors limit optimal therapeutic efficacy of TIL-ACT including that tumor-specific T cells are typically found in an exhausted state at baseline (i.e., altered or reduced effector function, high expression of multiple inhibitory receptors, reduced proliferative capacity, etc.) in the tumors of origin.[9] Although expansion in the presence of high-dose interleukin-2 (IL-2) results in TIL-ACT products in which tumor-specific clonotypes exhibit attenuated epigenetic or phenotypic features of exhaustion, the transferred TILs can readily reacquire features of exhaustion *in vivo* post ACT.[10] Exhaustion is therefore relevant in restraining TIL function and targeting checkpoints through genetic editing holds the potential to enhance their clinical efficacy.[11,12]

Previous preclinical studies have demonstrated the feasibility of using CRISPR-Cas9 technology to genetically modify TILs to improve their persistence and performance.[11–13] CRSIPR-Cas9, however, induces double-strand breaks (DSBs) in DNA and comes with the risk of improper translocations, inversions, and chromosomal deletions at sites of endonuclease activity, even more so in the context of multiplex-editing.[14–16] Advances in gene editing technologies such as base editors (BE) which precisely and efficiently generate targeted point

Received 1 October 2024; accepted 20 August 2025;
https://doi.org/10.1016/j.omton.2025.201041.

Correspondence: Morteza Hafezi, Ludwig Institute for Cancer Research, Department of Oncology, University of Lausanne and Lausanne University Hospital (CHUV), Lausanne, Switzerland.
E-mail: morteza.hafezi@unil.ch
Correspondence: Melita Irving, Ludwig Institute for Cancer Research, Department of Oncology, University of Lausanne and Lausanne University Hospital (CHUV), Lausanne, Switzerland.
E-mail: melita.irving@unil.ch
Correspondence: George Coukos, Ludwig Institute for Cancer Research, Department of Oncology, University of Lausanne and Lausanne University Hospital (CHUV), Lausanne, Switzerland.
E-mail: george.coukos@chuv.ch

Published by Elsevier Inc. on behalf of The American Society of Gene and Cell Therapy.
This is an open access article under the CC BY license (http://creativecommons.org/licenses/by/4.0/).

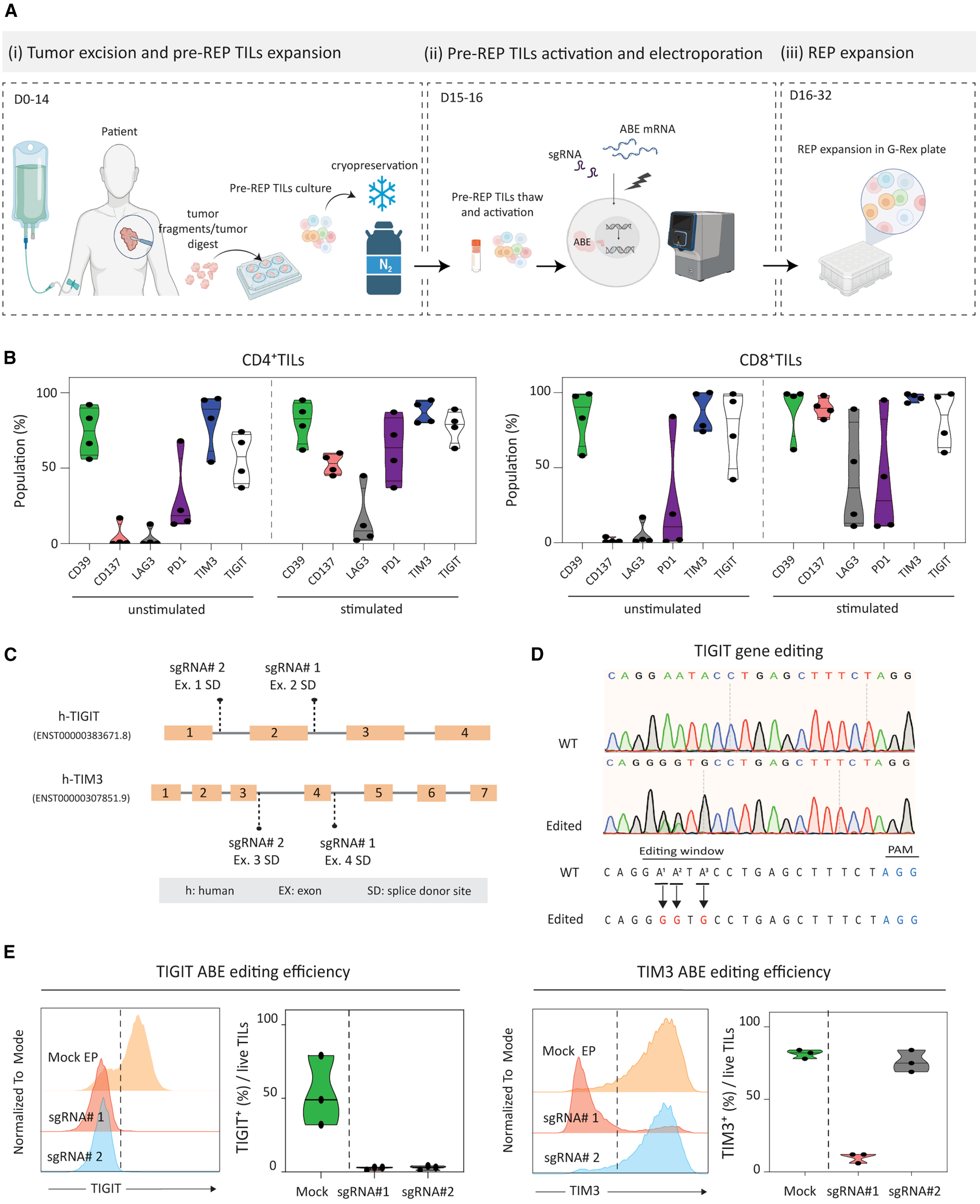

(legend on next page)

mutations without requiring DSBs like CRISPR-Cas9 lower the incidence of inducing harmful genetic rearrangements and may be a safer approach for knockouts (KO) in TILs and other T cell products in general.[17,18]

Here, we have developed a small-scale, good manufacturing practice (GMP)-compatible procedure for both single and dual ABE of TILs. We have further demonstrated proof-of-principle for efficient dual ABE KO of the co-inhibitory receptors TIM3 and TIGIT, which we found to be widely expressed in human melanoma and ovarian cancer-derived TILs. We observed that dual TIM3 and TIGIT KO in TILs augmented fold-expansion during the rapid expansion protocol (REP) as well as effector functions including cytokine production and serial killing upon co-culture with autologous patient-derived tumor cells. Finally, the edited TILs were readily able to penetrate patient-derived spheroids and confer long-term control of patient-derived tumors *in vivo*. Overall, our study demonstrates the feasibility and safety of editing TILs by ABE and indicates that dual KO of TIM3 and TIGIT in TILs derived from both melanoma and ovarian cancer warrants further investigation for potential clinical translation.

RESULTS

Feasibility of adenine base editing for TIL ACT workflow

To assess the feasibility of BE for TIL engineering, we implemented ABE to target co-inhibitory receptors in TILs from ovarian (Ova-TIL) or melanoma (Mel-TIL) patients expanded with high dose IL-2 by conventional REP (schematic in Figure 1A). We chose ABE as opposed to cytosine base editing (CBE) because the former has been shown to be more efficient and safer for T cell engineering purposes.[19] We began by screening the exhaustion profiles of REP-expanded (i.e., activated and cultured in the presence of high-dose IL-2) Ova- and Mel-TILs derived from different patients. A relative low proportion of the REP-TILs expressed PD1 and LAG3, but a high proportion of both $CD4^+$ and $CD8^+$ REP-TILs expressed CD39, TIM3, or TIGIT (Figures 1B and S1). The proportion of REP-TILs expressing these markers were either maintained (CD39) or upregulated (TIM3 and TIGIT) upon re-stimulation for 24 h with anti-CD3/CD28 beads (Figure 1B). In addition, we observed that both irradiated feeder cells and autologous patient-derived tumor cell lines expressed elevated levels of TIM3 ligand galectin-9, and TIGIT ligands CD112 and CD155 (Figures S2 and S3). Hence, both TIM3 and TIGIT are relevant candidates for TIL editing and functional analyses.

Initially, we assessed ABE of single genes across different patient TIL samples. Briefly, ABE-matched gRNAs were designed (Table S1) to target the donor or acceptor splice sites of the TIM3 or the TIGIT locus (Figure 1C). Frozen pre-REP Ova-TILs and Mel-TILs were thawed and activated for two days in serum-free medium supplemented with IL-2 and anti-CD3 monoclonal antibody and subsequently electroporated (EP) using the MaxCyte ExPERT ATx device with ABE mRNA and guide (g)RNA. The EP TILs were then transferred to G-Rex plates containing serum-free medium supplemented with IL-2, anti-CD3 antibody and irradiated feeder cells for the REP phase of TIL expansion. At the end of the REP phase we assessed KO efficiency of TIM3 or TIGIT for the different gRNAs. We confirmed adenine (A) to guanine (G) conversion by Sanger sequencing at the A nucleotide in TIM3 and TIGIT protospacer sequences (Figures 1D and S4). The single ABE with the best gRNAs resulted, on average, in ~95% KO of TIGIT and ~88% KO of TIM3, as measured at the protein level in both Ova- and Mel-TILs (Figure 1E). We chose TIGIT gRNA #1 and TIM3 gRNA #1 for dual gene-editing as they exhibited the highest gene KO efficiency.

Highly efficient dual TIM3- and TIGIT-knockout in TILs by adenine base editing

Toward our goal of editing both TIM3 and TIGIT in TILs, we next co-delivered the best splice-disrupting gRNAs from the initial screening to engineer dual KO TILs. We generated up to 93% (mean = 85%) and 97% (mean = 85%) dual KO of TIM3 and TIGIT in $CD4^+$ and $CD8^+$ TILs, respectively, as measured by cell-surface protein expression by flow cytometric analysis. We observed high KO efficiency in both unstimulated and stimulated $CD4^+$ and $CD8^+$ TILs (Figure 2A). Deep sequencing analysis confirmed that ABE-mediated deamination of the desired splice site of TIM3 and TIGIT resulted in up to 96% (A1 site, mean = 83%) and 94% (A3 site, mean = 87%), respectively, conversion of A·T pairs to G·C pairs within the editing window (Figure 2B). ABE nickase activity should mitigate insertion-deletion mutations (indels) which frequently occur upon CRISPR-Cas9-induced DSBs. To test this, we interrogated edited TILs by next-generation sequencing (NGS) to quantify indel frequencies at ABE target sites (Figure 2C). NGS analysis showed that ABE-mediated splice

Figure 1. Adenine base editing for TIL engineering

(A) Schematic overview of the study design. (1) After resection, tumor lesions were minced into small fragments. For the Pre-REP, tumor fragments were either digested or cultured in medium supplemented with high dose of IL-2, or directly seeded to allow the outgrowth of TILs. Pre-REP TILs were cryopreserved at this stage. (2) Frozen Pre-REP TILs were thawed and activated for two days. Activated TILs were electroporated (EP) with the desired combination of adenine base editor and guide (g)RNA using the MaxCyte ExPERT ATx device. (3) For the REP, one hour after EP the TILs were transferred to a 24-well G-Rex plate with serum-free medium supplemented with anti-CD3 (OKT3) monoclonal antibody, IL-2, and irradiated feeder cells. The phenotype and function of Mock-EP and ABE-TILs were analyzed at the end of the REP protocol. (B) Screening of activation and exhaustion markers in unstimulated and post-REP TILs stimulated for 24 h with anti-CD3/CD28 beads. Both $CD4^+$ and $CD8^+$ subsets of two Ova- and two Mel-TILs post-REP were analyzed (n = 4). (C) Diagram of TIM3 and TIGIT locus showing relative positions for splice-disrupting gRNAs. (D) Confirmation of A to G conversion in edited TILs. Chromatograms obtained by Sanger sequencing demonstrate the point mutation introduced by ABE in exemplary TIGIT-edited TILs. (E) Engineering single gene KO TILs. Exemplary histogram for TIM3 and TIGIT expressions in Mock-EP and ABE Ova-and Mel-TILs. Violin plots show quantification of KO efficiency in REP-TILs (n = 3). Mock-EP TILs were transfected with ABE mRNA alone and were used as a control in all experiments. The Illustration was created with BioRender.com.

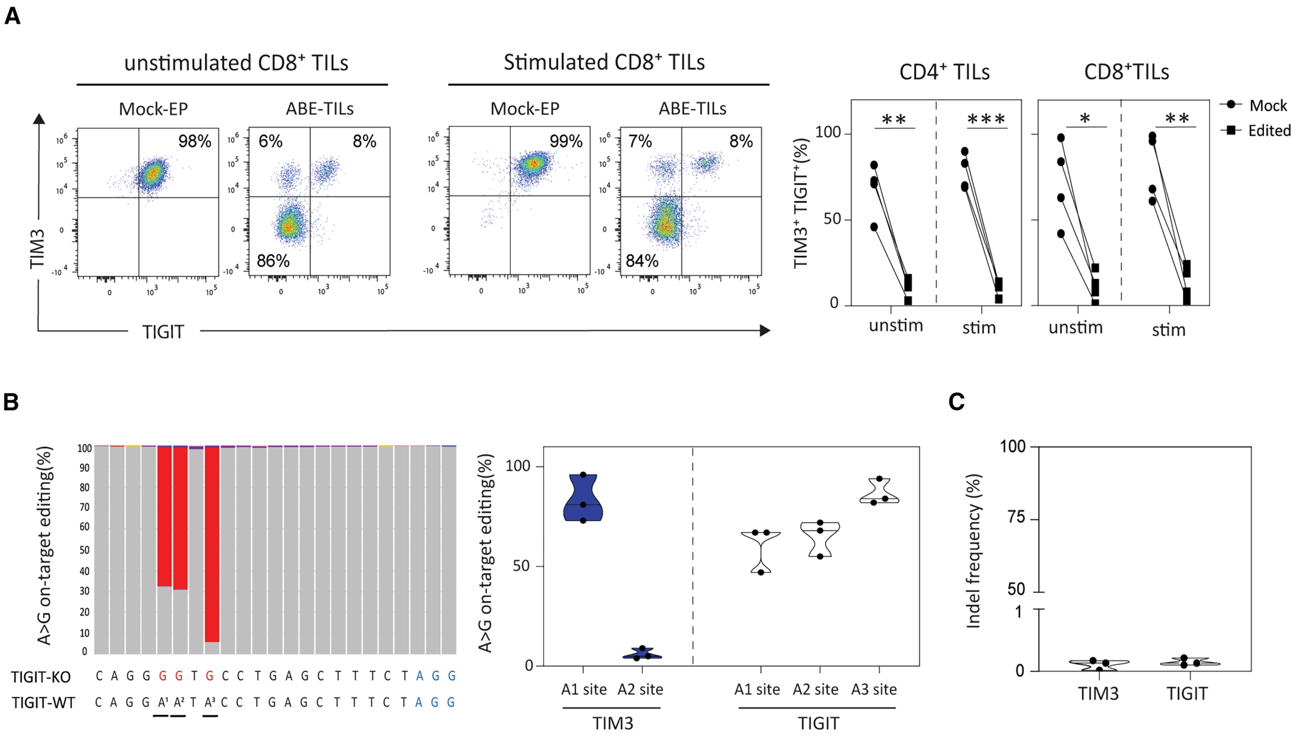

Figure 2. Optimization of TIL double gene editing using adenine base editing

(A) Engineering of double gene KO TILs. Left: exemplary dot plot shows double KO efficiency in TILs. Right: bar plot shows double KO efficiency in $CD4^+$ and $CD8^+$ TILs (n = 4). stim: stimulated; unstim: unstimulated. (B) Quantification of A to G conversion for various adenine site within the detected editing window of both TIM3 and TIGIT locus by deep sequencing (n = 3). The adenine mutation is shown in red in a bar graph. (C) Quantification of indel frequency at TIGIT and TIM3 target site analyzed by NGS (n = 3). Mock-electroporated T cells were transfected with ABE mRNA alone and were used as a control in all experiments. For Statistical analysis paired t test was performed. Statistical significance indicated as $^*p < 0.05$, $^{**}p < 0.01$ and $^{***}p < 0.001$.

disruption generated on average 0.1% and 0.14% indels at the target site of TIM3 and TIGIT, respectively. Further, a side-by-side comparison of edited TILs confirmed that the in-frame and out-of-frame indels occurring upon CRISPR-Cas9 editing were largely mitigated using ABE (Figures S5 and S6).

Phenotypic and functional analysis of TIM3-and TIGIT-edited TILs

Next, we sought to determine whether ABE-edited TILs (ABE-TILs) retain their phenotype and key effector functions. Phenotypic analysis of resting and (antigen-independent) activated TILs revealed no major changes in the activation and exhaustion profile of mock electroporated (Mock-EP) versus ABE-TILs (Figures 3A, S7, and S8). Interestingly, all Mel-TILs at steady state highly express DNAM-1 (CD226) (Figure S9), a surface protein that can provide costimulation in T cells upon engagement of CD112 or CD155.

As expected, the co-delivery of ABE mRNA and gRNAs led to a reduction in TIL viability compared to Mock-EP TILs as measured by cell counts immediately after EP (Mock-EP:63% versus ABE-+gRNA-EP: 47%, Figure S10). Despite the initial decrease in TIL numbers, the subsequent fold-expansion of single and dual gene-edited TILs significantly improved compared to Mock-EP ones (Figures 3B and S8C).

Phenotypic evaluation of Mock-EP and ABE-TILs revealed no major change in the $CD4^+/CD8^+$ ratio or differentiation markers in both Mel-TILs and OVA-TILs, with the main proportion having an effector memory (TEM) phenotype. (Figures 3C and 3D). With respect to effector functions, both Mock-EP and ABE-TILs exhibited no significant differences in the proportion of TNF-α or IFN-γ producing cells in $CD4^+$ and $CD8^+$ subsets upon non-specific activation with anti-CD3/CD28 beads (Figure 4A). Notably, however, the frequency of TNF-α, and IFN-γ producing T cells was increased for ABE-TILs upon co-culture with autologous tumor lines naturally expressing TIGIT or TIM3-specific ligands (i.e., CD112 and CD155 and galectin-9, respectively) (Figures 4B and S3). In terms of cytolytic capacity, although ABE-TILs showed minimal improvement in initial challenge, they demonstrated superior serial tumor-cell killing capacity as compared to Mock-EP TILs (Figures 4C, 4D, S8, and S11). Evaluation of exhaustion markers in TILs following tumor-cell rechallenge revealed a slight decrease in the frequency of $LAG3^+$ cells, but no changes in $PD1^+$ or $CD39^+$ expression were observed in ABE-TILs (Figure 4E).

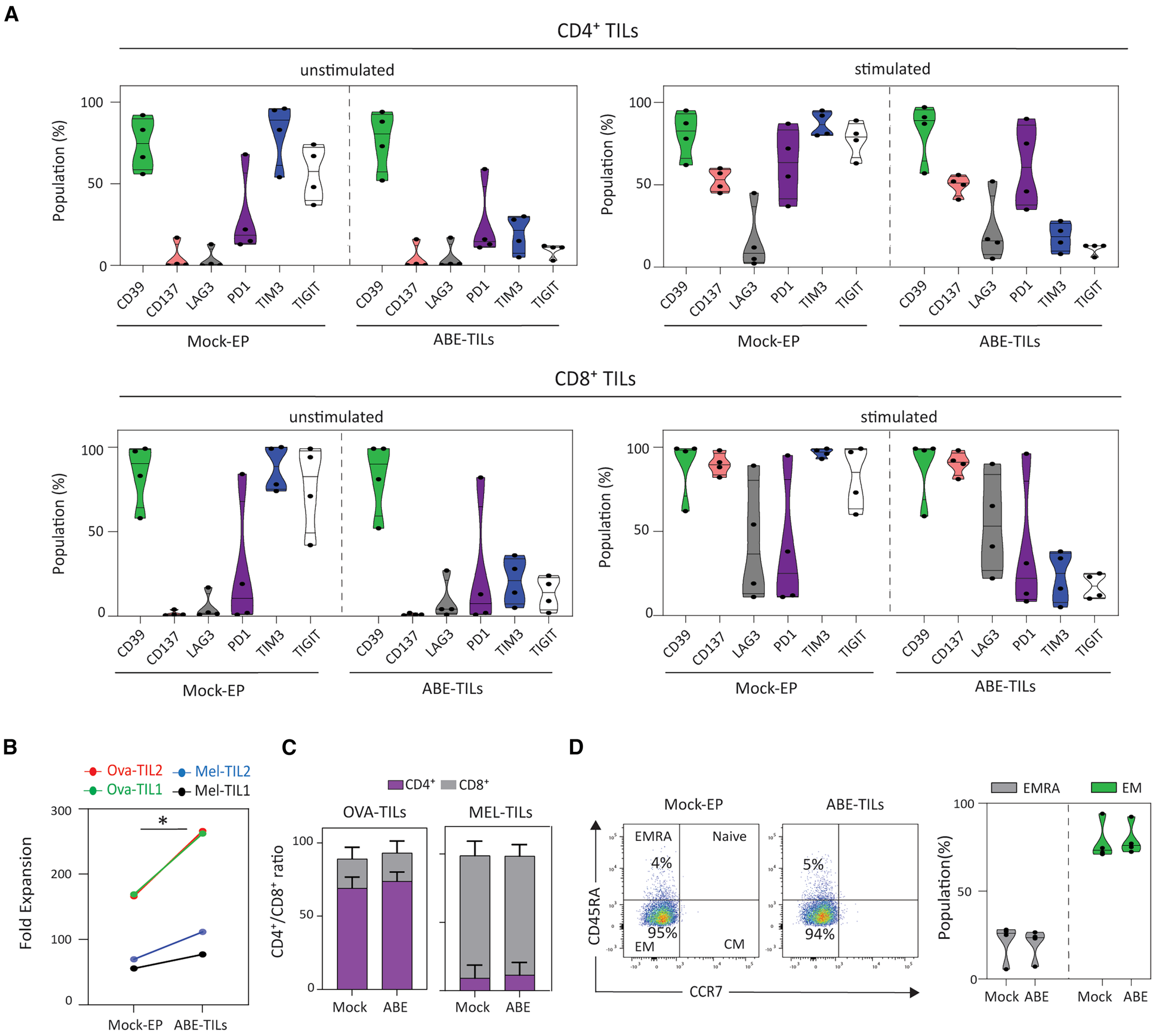

Figure 3. Dual adenine base editing of TIM3 and TIGIT enhances the fold-expansion of TILs during REP without altering phenotype

(A) Immunophenotyping of ABE-TILs. Violin plots demonstrate activation/exhaustion markers in resting and stimulated $CD4^+$ and $CD8^+$ subset of Ova- and Mel-TILs. (B) Fold-expansion was calculated at the end of REP phase for ABE-TILs and Mock-EP TILs (n = 4). TIL numbers immediately after electroporation used as the initial count. (C) $CD4^+$/$CD8^+$ T cell ratio in ABE- and Mock-EP TILs. (D) Phenotypic analysis of ABE-versus Mock-EP TILs. (TN: $CCR7^+$ $CD45RA^+$, TEM: $CCR7^-$ $CD45RA^-$, TCM: $CCR7^+$ $CD45RA^-$, TEMRA: $CCR7^-$ $CD45RA^+$ (n = 4). TN: naive T cells, TEM: effector memory T cells, TCM: central memory T cells, TEMRA: terminally differentiated effector memory T cells re-expressing CD45RA. For statistical analysis paired t test was performed. Statistical significance indicated as *$p < 0.05$.

Two-dimensional (2D) monolayer culture systems lack key features of three-dimensional (3D) tumor architecture and cannot be used to evaluate T cell infiltration. Hence, we next generated melanoma patient-derived spheroids (Figures 5A and S12). Following co-culture with the spheroids, histological and fluorescence microscopy analysis revealed that both Mock-EP and ABE-TILs effectively infiltrated the spheroids over time and were able to kill and disrupt the 3D structure of the spheroids (Figures 5B and S12).

Finally, we sought to test the performance of dual ABE-TILs *in vivo*. To that end, we performed a Winn assay in which NSG mice were subcutaneously co-injected with autologous patient-derived melanoma cells and Mock-EP or dual ABE-TILs. ABE-TILs exhibited superior control of tumor outgrowth and conferred improved overall survival in the Winn assay. Furthermore, there were no signs of graft-versus-host disease (GvHD) in any of the treated mice (Figures 6 and S13).

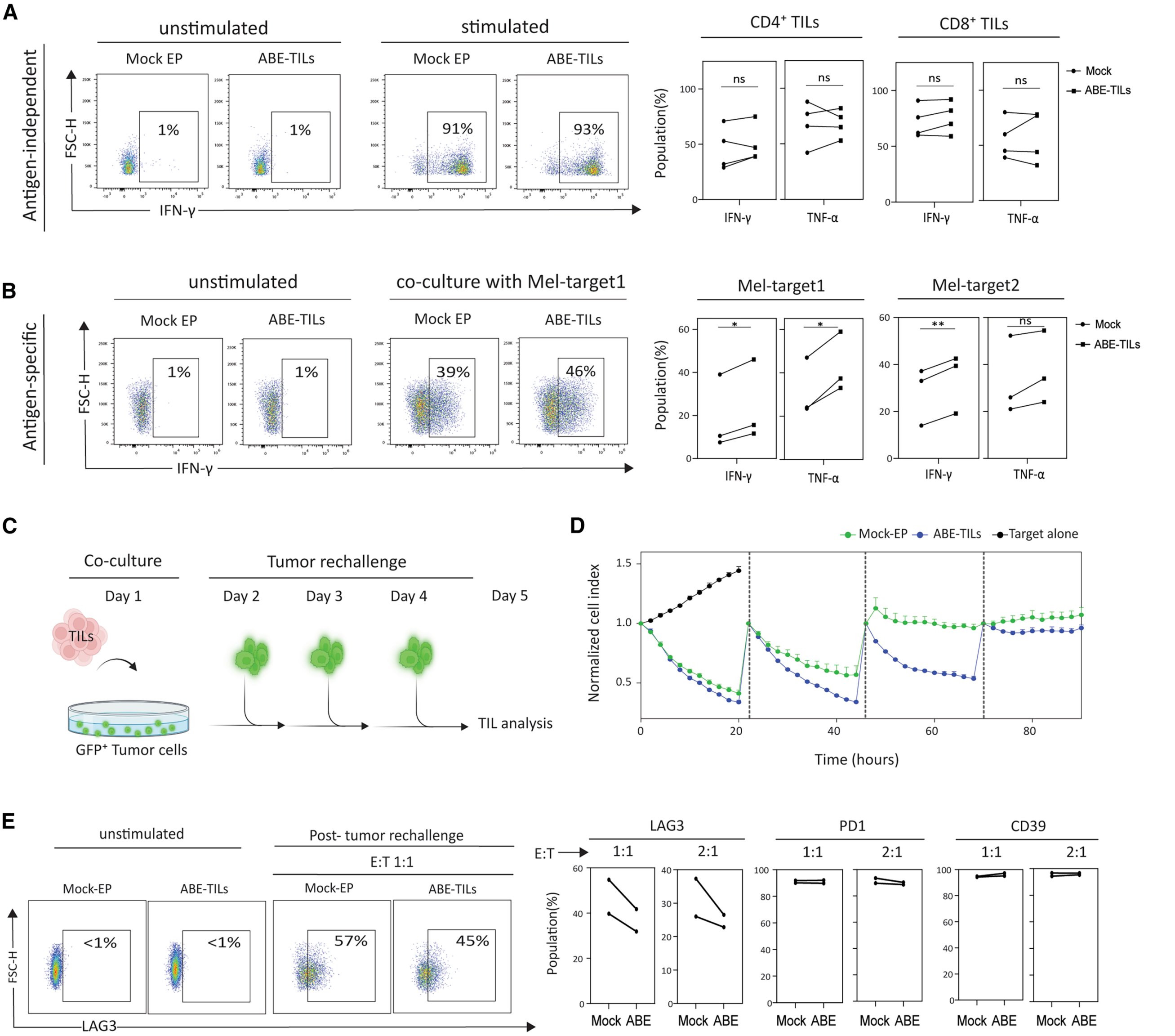

Figure 4. Dual adenine base editing of TIM3 and TIGIT improves cytokine production and serial killing by TILs

(A) Left: exemplary plots show the percentage of IFN-γ producing TILs activated with anti-CD3/CD28 beads. Right: Bar plots represent pooled data from 4 different TILs. (B) Left: exemplary plots show the percentage of IFN-γ producing TILs co-cultured with Mel-traget1. To evaluate tumor-specific effector function, REP-TILs were co-cultured at an effector-to-target ratio of 1:1 with autologous melanoma target cells overnight. Intracellular IFN-γ and TNF-α were measured using flow cytometry. Right: Bar plots represent pooled data from 3 independent experiments for each Melanoma-specific TIL. (C) Experimental design for tumor rechallenge experiment. The Illustration was created with BioRender.com. (D) Exemplary plot showing serial cytolytic ability of T cells determined by a real-time killing assay. The plot is exemplary of 3 independent experiments. Edited and non-edited TILs were co-cultured at an effector-to-target ratio of 2:1 with autologous melanoma target cells. T cell-mediated cytotoxicity was determined by measuring GFP positive tumor cells at 2-h intervals for approximately 90 h post T cell addition using the Incucyte S3 system. (E) Evaluation of exhaustion markers in TILs after repeated tumor challenge. Each plot represents pooled data from 2 independent experiments for melanoma-specific TIL. Mock-electroporated T cells were transfected with ABE mRNA alone and were used as a control in all experiments. For Statistical analysis paired t test was performed. Statistical significance indicated as *$p < 0.05$ and **$p < 0.01$. ns; not significant.

DISCUSSION

Successful and durable patient responses to TIL therapy strongly correlates with T-lymphocyte persistence and function post-ACT.[20] CRISPR-Cas9 gene KO of cell-surface expressed or intracellular immune checkpoints is a promising strategy for improving the efficacy of TIL-ACT.[11,13,21] However, DSBs induced in the genome by Cas9 can give rise to genetic alterations in the final T cell product[22–24] that could cause lymphoproliferative diseases in treated patients.[25]

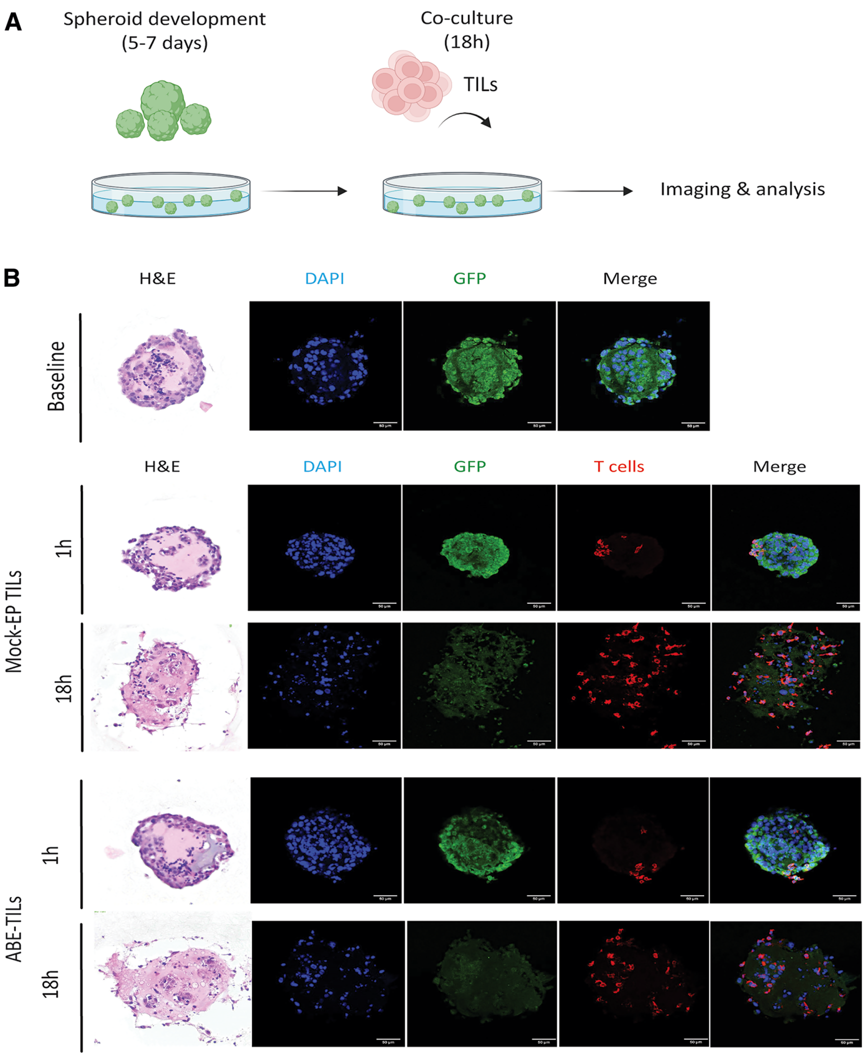

Figure 5. Dual TIM3 and TIGIT adenine base edited TILs readily penetrate spheroids and kill tumor cells

(A) Experimental design for melanoma spheroid development and tumor challenge. (B) Exemplary images of immunofluorescence (IF) and hematoxylin and eosin (H&E) staining of tumor spheroids at baseline and post co-culture with TILs. Mock-EP and ABE-TILs were co-cultured at an effector-to-target ratio of 1:1 with melanoma spheroids and T cell-mediated cytotoxicity and infiltration was determined over 18 h of co-culture. Baseline Images show the initial state of the spheroids. (DAPI: nuclei; red: $CD8^+$; GFP: tumor; Scale bars indicate 50 μm). Data and images are exemplary of two independent experiments.

Adenine and cytosine base editors, whereas, only induce single-strand DNA breaks which strongly mitigates the likelihood of significant genetic aberrations[18] and makes them more appealing tools for gene KO in T cells for clinical use.

In our study, we have established a GMP-compatible ABE protocol for TILs and evaluated the strategy for both ovarian and melanoma cancer-derived TILs. First, we showed that ABE can be readily integrated into the TIL-ACT workflow. In our small-scale setup, a low number of TILs ($1\text{-}4\times10^6$ TILs) obtained from pre-REP samples were edited using EP and subsequently expanded by REP. The small-scale EP strategy was optimized using the MaxCyte ExPERT ATx device for facile scalability to GMP-production and clinical translation. Notably, the starting pre-REP TILs used for editing were thawed from cryopreserved samples and the editing process was set-up with a low number of TILs in serum-free medium, all factors which can be limiting to the efficient production of an engineered TIL product. Indeed, in general TILs represent a more fragile and sensitive starting material than peripheral blood T cells, and beginning with larger numbers of fresh TIL samples or/and culturing them for editing in the presence of serum would likely augment their ease of production. However, we opted to use serum-free medium to increase safety and reproducibility of GMP manufactured ABE TIL products for clinical use.

With our method, we demonstrated highly efficient dual KO of TIM3 and TIGIT in both $CD4^+$ and $CD8^+$ TILs. Deep sequencing analysis confirmed that ABE of both Mel-TILs and Ova-TILs specifically converted A·T pairs to G·C pairs in the editing window of the corresponding splice site. One key difference between native Cas9 and modified nCas9 use for BE, which we refer to as a nickase, is that DSBs generated by the former are repaired mainly by the low fidelity non-homologous end-joining (NHEJ) and often cause a high frequency of indels at the resection site which, as mentioned, could cause genetic aberrations.[16] In contrast, nickase activity should circumvent indels taking place at the editing site of the gene. In line with the previous reports for peripheral blood T cells,[16,17] we observed a low level of indel events in ABE-as compared to CRISPR-Cas9- edited TILs, making it more desirable approach for multiplex editing.

ABE did not alter TIL phenotype, the $CD4^+/CD8^+$ ratio, or the general profile of activation and exhaustion markers. Although we observed an initial decline in ABE-TILs following EP as compared to Mock-EP TILs (probably due to toxicity upon mRNA delivery), this effect was transient. At the end of the REP phase the dual TIM3 and TIGIT ABE-TILs exhibited significantly higher fold-expansion, in line with previous reports demonstrating that blockade of TIM3 or TIGIT improves T cell proliferation.[26,27] In our study, we observed that the irradiated feeder cells used for REP expansion express CD112, CD155, and secrete galectin-9, all of which are known to inhibit

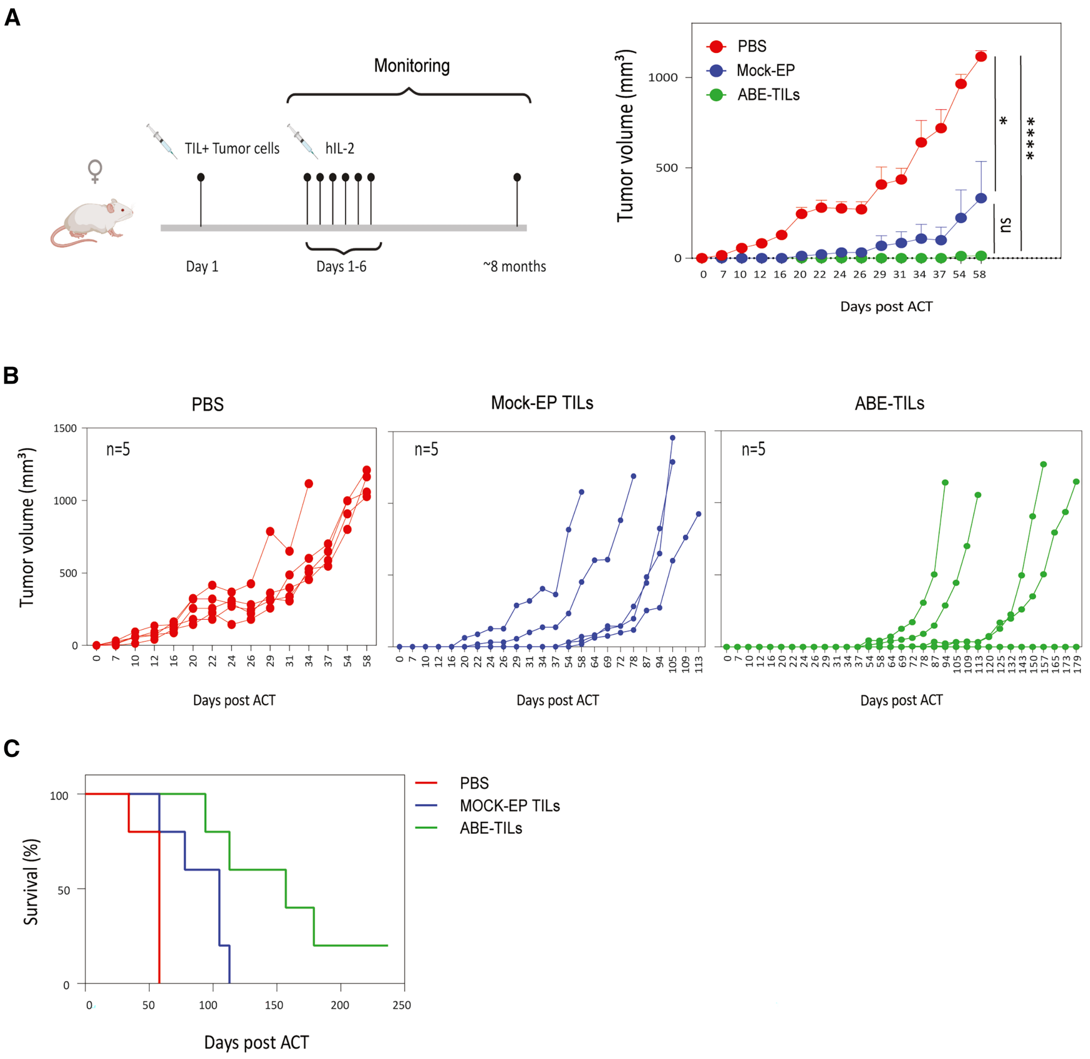

Figure 6. Adenine base edited TILs control tumor outgrowth in NSG mice and prolong their survival

(A) Top left: experimental set-up for the Winn assay. Top right: control of tumor outgrowth curves (means ± SEM, PBS = 5 mice, Mock-electroporated (EP) TILs = 5 mice, edited TILs = 5 mice). (B) Control of tumor outgrowth curves for individual mice in each group. (C) Kaplan-Meier survival curves for NSG mice treated with Mock-EP and ABE-TILs. PBS-treated mice were used as the negative control group. Statistical analysis for (A) was performed by two-way ANOVA with Tukey's multiple comparison tests. Statistical significance indicated as *$p < 0.05$, and ****$p < 0.0001$. ns; not significant. The Illustration in (A) was created with BioRender.com.

T cell proliferation.[26,28] By knocking out TIM-3 or/and TIGIT receptors, we rendered TILs resistant to their corresponding ligands, ultimately improving their expansion during REP. On the other hand, the TILs highly express DNAM-1 and hence may benefit from costimulation during REP upon engagement of CD112 and CD155 on the feeder cells when TIGIT is edited and no longer competes for binding. These findings suggest that inhibitory molecules released by feeder cells can limit TIL growth, and that blocking or editing these pathways could be a beneficial strategy to optimize REP-TIL manufacturing.

Notably, a higher proportion of dual ABE-TILs than Mock-EP TILs produced IFN-γ and TNF-α upon co-culture with autologous target tumor cells. It has been shown that the interaction between TIM3 and TIGIT with their specific ligands is inhibitory to cytokine production and immunotherapy response in melanoma.[26,29] In this regard, improved cytokine production in ABE-TILs could be explained by the natural expression of TIM3 or TIGIT-specific ligands in melanoma tumor lines which can only suppress the Mock-EP TILs. A recent report also revealed an intriguing role for TIM3 as a growth suppressive receptor in melanoma cells, implying that systemic blockade of TIM3 along with TIL-ACT may offer limited benefits as compared to TIM3 KO in TILs for treating melanoma patients.[30] Interestingly, dual KO and TIGIT KO TILs demonstrated higher serial killing capacity than TIM3 KO TILs. This may be

explained by the higher expression of TIGIT-compared to TIM3-specific ligands in the autologous patient-derived melanoma line used in this study. Moreover, TILs also express DNAM-1 a costimulatory receptor that binds CD112 and CD155 but is outcompeted by TIGIT when they are coexpressed. Hence, KO of TIGIT not only blocks inhibitory signaling but may also enable costimulatory signaling via DNAM-1. This hypothesis requires further investigation with additional TIL samples and autologous patient-derived tumor cell lines. Finally, we demonstrated successful infiltration of ABE-TILs into patient-derived tumor spheroids and control of tumor outgrowth *in vivo* without signs of GvHD in the treated mice.

In summary, to the best of our knowledge, we have described the first application of ABE for TIL engineering. Our protocol is readily GMP-adaptable and highly efficient. Taken together, our data demonstrate the feasibility and improved safety of ABE as compared to CRISPR-Cas9 KO in TILs. In future studies, other immune checkpoints will be targeted alone and in combination with TIGIT and the different ABE-TILs tested against matched patient-derived tumor cell lines. We conclude that ABE of immune checkpoints in TILs is a promising strategy for safely improving patient responses in the clinic.

MATERIALS AND METHODS

Human samples, pre-rep TIL and tumor cell line development

Melanoma pre-REP TILs were generated from fresh or cryopreserved tumor specimen using a GMP-compliant manufacturing process, as previously described.[31] Ovarian pre-REP TILs were obtained from cryopreserved total dissociated samples following plating in RPMI (BioConcept) supplemented with 8% human serum (Biowest) and 6000 IU/mL human IL-2.[32] Autologous melanoma tumor cell lines (TCLs) were generated as previously described.[10,31] Mel-target1 and Mel-target3 cells were cultured in R10 medium comprising RPMI-1640 with 10% fetal bovine serum (FBS) and 1% penicillin/streptomycin at 37°C with 5% CO^2. Mel-target2 cells were cultured in medium containing 70% of RPMI-1640 (BioConcept) with 10% FBS (Gibco) and 1% penicillin/streptomycin (BioConcept) and 30% complete melanocyte growth medium (PromoCell) at 37°C with 5% CO^2. All procedures were performed under protocols approved by the respective institutional regulatory committees at the University of Pennsylvania, USA, and the University of Lausanne and Lausanne University Hospital (CHUV), Switzerland. Informed consent was obtained from all patients.

Guide RNA design

BE splice disrupting guide RNAs (gRNAs) were designed by SpliceR program (https://z.umn.edu/splicer) and purchased from Synthego. Ensemble transcript ID of TIM3 (ENST00000307851.9) and TIGIT (ENST00000383671.8) were used to obtain ABE-matched gRNAs. Sequences of the gRNAs are listed in Table S1.

ABE mRNA electroporation and TILs rapid expansion protocol

ABE 8.20 mRNA plasmid was purchased from Addgene and *in vitro* transcribed using the mMESSAGE mMACHINE T7 ULTRA transcription kit (Thermo Fisher Scientific), according to the manufacturer's instructions. For electroporation, frozen pre-REP TILs thawed and activated in serum-free X-VIVO15 supplemented with 3000 IU/mL of recombinant human IL-2 (Proleukin, Novartis, Basel, Switzerland) and 30 ng/ml GMP-grade soluble anti-CD3 (OKT3) antibody (Miltenyi Biotech) for 1–2 days before electroporation. Activated TILs were washed with Opti-MEM reduced serum medium (Gibco) prior to resuspension in MaxCyte buffer (HyClone). For single and dual editing, $1\text{-}4\times10^6$ Pre-REP TILs were mixed with 2 μg of ABE8.20 mRNA and 3 μg of chemically modified ABE-matched guide RNA (Synthego), then topped up to 25 μL with MaxCyte buffer. The mixture was transferred to a processing assembly (OC-25 × 3) (MaxCyte) and electroporated using the Expanded T Cell-3 protocol on the MaxCyte ExPERT ATx device. Following electroporation, the processing assembly was incubated at 37°C for 15 min. Initial count was performed by hemocytometer (Marienfeld) and 0.1% Tryphan blue (Gibco). After counting, 1×10^6 TILs were transferred to a G-Rex 24-well plate (Wilson Wolf) containing X-VIVO15 supplemented with 3000 IU/mL of human IL-2, 30 ng/mL of GMP-grade soluble CD3 antibody (OKT3), and 1×10^8 irradiated allogeneic PBMC feeder cells (TIL-to-feeder ratio of 1:100). On day 7 post-REP, all wells were refreshed with X-VIVO15 supplemented with 3000 IU/mL of human IL-2. Between days 14 and 17, REP TILs were collected and used for downstream analysis. All experiments were conducted at the Lausanne Branch of the Ludwig Institute for Cancer Research (LLB).

Sanger and next-generation sequencing analysis

Genomic DNA (gDNA) was extracted using the Quick-DNA Miniprep Plus Kit (Zymo Research) according to the manufacturer's instructions. The gDNA was then used to amplify the region targeted by the guide RNA using primers flanked with Nextera adapter sequences (Illumina), provided in the supplemental data (Table S2). The amplified PCR products were purified and quantified using a Qubit instrument (Thermo Fisher). To confirm ABE editing, the purified PCR products were checked by Sanger sequencing and analyzed using the web app EditR (baseeditr.com).[33] For deep NGS analysis, 2 ng of the product were used to perform a second PCR in which Illumina indexes were added for multiplexing. Libraries were purified with AmpureXP beads (Beckman Coulter), quantified with Qubit (Thermofisher) and sequenced on Illumina instruments using paired-end run. Sequences were aligned against the reference using BWA and Samtools algorithms. Nucleotide frequencies as well as indels were then extracted using IGVtools. The size of indels was calculated using an ad hoc perl script to determine whether indel have created a frameshift.

Phenotypic analysis of TIL feeder cells and melanoma cells

For TIL immunophenotyping, post-REP TILs were non-specifically activated with Dynabeads Human T-Activator CD3/CD28 beads (Thermo Fischer Scientific) at 2:1 bead-to-cell ratio for 24 h. This was followed by staining of various activation and exhaustion surface markers including, anti-human CD3-PE, CD4-PerCP-Cy5.5, CD8-AF488, CD8-APC, TIM3-PE, TIGIT-BV605, CD39-FITC,

PD1-BV421, CD137-BV650, LAG3-APC, and DNAM-1 for unstimulated or stimulated TILs. All the aforementioned antibodies were purchased from BioLegend. Fixable Near-IR dead cell stain kit (Thermo Fisher Scientific) was used to discriminate live and dead cells. The exemplary gating strategy for TIL immunophenotyping can be found in Figure S1. Anti-human CCR7-BV421 and anti-human CD45RA-APC were used to evaluate the differentiation status of TILs after REP expansion.

To evaluate the expression of TIGIT-specific ligands on melanoma tumor lines, cells were stained with antibodies targeting CD112 and CD155. All samples were acquired by LSR II flow cytometry machine and further analyzed with FlowJo 10.7.2 software.

Measurement of cytokine production by TILs

For intracellular cytokine staining (ICS) analysis, TILs were activated either by Dynabeads (antigen-independent) or tumor-specific lines (antigen-dependent). For antigen-independent activation, anti-CD3/CD28 dynabeads were used at bead-to-cell ratio of 2:1 in round-bottom 96-well plate for 5 h in X-VIVO15 medium containing brefeldin A (BD GolgiPlug, 1:1,000), and Monensin (BD GolgiStop, 1:1,000) (BD Biosciences). For tumor-specific activation, TILs were co-cultured overnight with autologous melanoma target cells at an effector-to-target ratio of 1:1 in X-VIVO15 medium supplemented with brefeldin A (BD GolgiPlug, 1:1,000) and Monensin (BD GolgiStop, 1:1,000) in a flat-bottom 96-well plate. Cytokine production by TILs was evaluated by intracellular staining of TNF-α, IFN-γ. Non-activated TILs were used as control in all experiments. To evaluate the expression of TIGIT and TIM3 specific ligands, melanoma target cells were stained with CD112-PE, CD155-APC, and galectin-9-PE, respectively. Unstained cells and/or stained isotype controls were used as controls for analyses. The antibodies were purchased from BioLegend. All samples were acquired by LSR II flow cytometry machine and further analyzed with FlowJo 10.7.2 software.

ELISA assay for measuring human galectin-9

Human galectin-9 levels were quantified using an ELISA kit (Quantikine ELISA, Biotechne, R&D Systems, DGAL90) according to the manufacturer's protocol. Briefly, supernatants were collected from target tumor cell cultures, or feeder cells four days post-irradiation. Supernatants were stored at −80°C until the assay. Both undiluted and diluted samples were tested, with culture media serving as the negative control.

Real time killing assay

T cell-mediated killing was measured by Incucyte S3 live-cell analysis (Essen Bioscience). Briefly, 20×10^3 GFP^+ melanoma target cells were seeded in 96 flat-bottom well plate wells (Costar, Vitaris) to adhere overnight. Mock-EP and ABE-TILs were added at an effector-to-target ratio (E:T) of 1:1 and 2:1 in serum-free X-VIVO 15 medium (Lonza). For tumor rechallenge, 20×10^3 GFP^+ melanoma target cells were added to the wells on days 1, 2, 3 post co-culture. Target cell killing was measured after co-culture by monitoring GFP objects at 2 h interval. Target killing analysis was performed using the Incucyte software provided by Essen Bioscience. The Normalized cell index (NCI) was determined by dividing the cell indices (total GFP^+ count) at each time point by those at the initial time point. Alternatively, for non-GFP^+ target cells, TILs were co-cultured at E:T of 1:1 with target cells in the presence 125 nM cytox red (Essen Bioscience). In this case, target death was quantified by measuring CytoX-Red positive cells at 2 h interval after co-culture. The total red object area per well was acquired by using the same Incucyte software as described earlier.

Spheroid generation

GFP^+ melanoma cells were seeded in a microcavity plate (SUN bioscience) at a density of 15,000 cells per well to ensure homogeneous growth. The cells were allowed to settle and aggregate at the bottom of the plate at 37°C in the presence of 5% CO^2 for at least 30 min. Afterward, R10 medium was added and cells were incubated at 37°C with 5% CO^2 for 5 to 7 days to develop spheroids of 200–300 μm diameter. All procedures followed the protocol provided by SUN bioscience for the Gri3D 96-well plate.

Co-culture of TILs and tumor spheroids

GFP^+ spheroids were harvested from the microwell plate and transferred to a ULA 96-well U-bottom plate (Sarstedt) for co-culture. To estimate the cell number in each spheroid, multiple microwells were collected, dissociated into single cells, and counted to quantify the number of cells per well and per spheroid. For the co-culture, cells were seeded at an effector-to-target ratio of 1:1 and incubated overnight at 37°C with 5% CO^2 in R10 medium. Images were captured at various time points using a brightfield microscope with a 5× objective. Following 18 h co-culture, spheroids were washed once with PBS and then fixed with 200 μL of 4% PFA at room temperature for 30 min in the dark. After fixation, the spheroids were washed twice with PBS and carefully transferred to a 96-well flat-bottom plate (Corning) for embedding in FFPE. Prior to paraffin embedding, cells were embedded in histogel (Epredia HG-4000-012) at 4°C for 30 min to allow the histogel to polymerize. Finally, the spheroids within the histogel were embedded in paraffin and sectioned with the microtome at 4 μm for hematoxylin/eosin and immunofluorescent staining.

H&E staining

To evaluate spheroid cytology and architecture, slides were dewaxed at 60°C on a pre-warmed heating plate for 15 min (Tech interlab) and deparaffinized in xylene (Reactolab) followed by rehydration through a graded ethanol (Reactolab) series. The slides were then immersed in distilled water and stained with hematoxylin staining (Avantor) for 4 min. Counterstaining was performed using acidic alcohol (Sigma), followed by rinsing with distilled water. For bluing, the slides were rested in lukewarm distilled water for 5 min, and then rinsed in distilled water. The slides were then stained by 0.2% eosin (Sigma) for 30 s and quickly rinsed in distilled water. Finally, the slides were dehydrated and mounted using one drop of Pertex (Biosystems). Images were acquired using a brightfield microscope (Nikon Inverted Routine Microscope ECLIPSE Ts2) with at 5× objective.

Immunofluorescent staining of spheroids

For immunofluorescence staining, FFPE spheroid samples were heated at 60°C on heating plate for 15 min (Tech interlab) and deparaffinized in pure xylene (Reactolab) followed by rehydration through a graded ethanol (Reactolab) series. Antigen retrieval was performed using Tris-EDTA at 95°C–100°C for 1.5 min. The samples were then cooled and washed in PBS. Following antigen retrieval, cells were permeabilized with 0.5% Triton X-100 (Sigma) in PBS for 10 min. To avoid non-specific binding, samples were incubated in blocking buffer (Sigma) at room temperature for 1 h. Cells were then stained with primary rabbit anti-CD8 antibody (CD Creative Diagnostic) followed by Alexa Fluor 555-conjugated anti-rabbit secondary antibody (Thermofisher). Finally, cells were counterstained with DAPI (Sigma) to determine the nuclei and mounted for fluorescence microscopy analysis. Images were acquired with Olympus FluoView 3000 at 40× objective.

Measurement of GFP expression levels and quantification of TIL infiltration in spheroids

Multiple fluorescent images of spheroids were acquired at different z-planes by confocal microscopy and analyzed with FIJI (ImageJ) software. GFP expression levels were quantified by defining a region of interest (ROI) along the inner perimeter of each spheroid. The spheroid areas were determined by staining nuclei with DAPI, which enabled accurate delineation of their boundaries. GFP fluorescence intensity within the ROI was measured and normalized to the spheroid area, yielding standardized GFP expression values in fluorescence intensity per square millimeter (GFP intensity/mm^2). For T cell ($CD8^+$ Mock-EP and ABE-TILs) infiltration analysis, cells were quantified using Alexa Fluor 555 channel. Manual counting was then conducted to distinguish $CD8^+$ T cells located inside the spheroids from those outside, allowing for an accurate assessment of infiltration. The density of T cells was subsequently normalized to the spheroid area, providing a measure of T cells per square millimeter ($CD8^+/mm^2$).

In vivo Winn assay

For the Winn assay, NOD-scid IL2Rgamma^{null} NSG mice aged 8–12 weeks were co-injected with TILs and autologous tumor cells. Briefly, non-edited (EP-Mock) and edited TILs (ABE-TILs) were mixed with melanoma target cells at an effector:target ratio of 3:1 (5×10^5 tumor cells + total 1.5×10^6 TILs) and subcutaneously inoculated into the right flank of the mice. Mice received 100,000 IU of human IL-2 (Proleukin, Novartis, Basel, Switzerland) subcutaneously for 6 consecutive days. Tumor growth was followed by caliper measurements 2–3 times a week for 26 days post injection. Tumor volume was calculated using the formula of $V = (L \times W2)/2$ (L = greatest longitudinal diameter and W = greatest transverse diameter). Upon TIL ACT and IL-2 injection, mice were carefully monitored for common clinical sign of GvHD including lethargy, hunching, fur loss, skin lesions, changes in activity level, and weight loss in accordance with regulatory guidelines.

Statistical analysis and data representation

Data analysis and visualization were performed by Prism (GraphPad, San Diego, CA) using relevant statistical tests as outlined in the figure legends (*$p < 0.05$; **$p < 0.01$; ***$p < 0.001$; ****$p < 0.0001$).

DATA AVAILABILITY

Raw data and analyses of all experiments presented in the article are available upon reasonable request.

ACKNOWLEDGMENTS

This work was generously supported by Ludwig Cancer Research, Cancera, and the Prostate Cancera, Biltema and Fondazione Teofilo Rossi di Montelera e di Premuda Foundations to G.C., as well as the Swiss National Science Foundation (SNSF# 310030_204326) and ISREC Foundation to M.I. We thank the MaxCyte team, in particular, Marie-Laure Joandel and Amandine Girard, for their support and contribution of consumables for EP to this project. We also thank Paula Aguilar Solana from Wilson Wolf Manufacturing for the provision of consumables.

AUTHOR CONTRIBUTIONS

M.H.: conceived and supervised the project, established ABE editing, performed experiments, analyzed data and wrote the manuscript. R.G., L.H., S.B., G.M.P.G.A., H.E.J., and L.P.: performed experiments and analyzed data. B.S.C.: performed sheroid experiments and analyzed data. T.M.: provided consumables and technical expertise for MaxCyte electroporation. M.A., D.C., B.G., and D.D.L.: provided technical expertise related to TILs or tumor sheroids. K.S.: provided reagents. A.H.: provided clinical material. M.I. and G.C. supervised the project, edited the manuscript and funded the work.

DECLARATION OF INTERESTS

G.C. has received grants and research support or has been co-investigator in clinical trials by Bristol-Myers Squibb, Tigen Pharma, Iovance, Hoffmann La Roche AG, and Boehringer Ingelheim. The Lausanne University Hospital (CHUV) has received honoraria for advisory services that G.C. has provided to Genentech, AstraZeneca AG and EVIR. G.C. has previously received royalties from the University of Pennsylvania for CAR-T cell therapy licensed to Novartis and Tmunity Therapeutics.

SUPPLEMENTAL INFORMATION

Supplemental information can be found online at https://doi.org/10.1016/j.omton.2025.201041.

REFERENCES

1. Muul, L.M., Spiess, P.J., Director, E.P., and Rosenberg, S.A. (1987). Identification of specific cytolytic immune responses against autologous tumor in humans bearing malignant melanoma. J. Immunol. *138*, 989–995.
2. Rosenberg, S.A., Packard, B.S., Aebersold, P.M., Solomon, D., Topalian, S.L., Toy, S.T., Simon, P., Lotze, M.T., Yang, J.C., Seipp, C.A., et al. (1988). Use of tumor-infiltrating lymphocytes and interleukin-2 in the immunotherapy of patients with metastatic melanoma. A preliminary report. N. Engl. J. Med. *319*, 1676–1680.
3. Dudley, M.E., Wunderlich, J.R., Robbins, P.F., Yang, J.C., Hwu, P., Schwartzentruber, D.J., Topalian, S.L., Sherry, R., Restifo, N.P., Hubicki, A.M., et al. (2002). Cancer regression and autoimmunity in patients after clonal repopulation with antitumor lymphocytes. Science *298*, 850–854.
4. Morgan, R.A., Dudley, M.E., Wunderlich, J.R., Hughes, M.S., Yang, J.C., Sherry, R.M., Royal, R.E., Topalian, S.L., Kammula, U.S., Restifo, N.P., et al. (2006). Cancer regression in patients after transfer of genetically engineered lymphocytes. Science *314*, 126–129.
5. Mullard, A. (2024). FDA approves first tumour-infiltrating lymphocyte (TIL) therapy, bolstering hopes for cell therapies in solid cancers. Nat. Rev. Drug Discov. *23*, 238.
6. Zacharakis, N., Huq, L.M., Seitter, S.J., Kim, S.P., Gartner, J.J., Sindiri, S., Hill, V.K., Li, Y.F., Paria, B.C., Ray, S., et al. (2022). Breast Cancers Are Immunogenic: Immunologic Analyses and a Phase II Pilot Clinical Trial Using Mutation-Reactive Autologous Lymphocytes. J. Clin. Oncol. *40*, 1741–1754.

7. Tran, E., Robbins, P.F., Lu, Y.C., Prickett, T.D., Gartner, J.J., Jia, L., Pasetto, A., Zheng, Z., Ray, S., Groh, E.M., et al. (2016). T-Cell Transfer Therapy Targeting Mutant KRAS in Cancer. N. Engl. J. Med. *375*, 2255–2262.

8. Tran, E., Turcotte, S., Gros, A., Robbins, P.F., Lu, Y.C., Dudley, M.E., Wunderlich, J.R., Somerville, R.P., Hogan, K., Hinrichs, C.S., et al. (2014). Cancer immunotherapy based on mutation-specific CD4+ T cells in a patient with epithelial cancer. Science *344*, 641–645.

9. Jiang, Y., Li, Y., and Zhu, B. (2015). T-cell exhaustion in the tumor microenvironment. Cell Death Dis. *6*, e1792.

10. Chiffelle, J., Barras, D., Pétremand, R., Orcurto, A., Bobisse, S., Arnaud, M., Auger, A., Rodrigo, B.N., Ghisoni, E., Sauvage, C., et al. (2023). Tumor-reactive clonotype dynamics underlying clinical response to TIL therapy in melanoma. Preprint at bioRxiv. https://doi.org/10.1101/2023.07.21.544585.

11. Fix, S.M., Forget, M.A., Sakellariou-Thompson, D., Wang, Y., Griffiths, T.M., Lee, M., Haymaker, C.L., Dominguez, A.L., Basar, R., Reyes, C., et al. (2022). CRISPR-mediated TGFBR2 knockout renders human ovarian cancer tumor-infiltrating lymphocytes resistant to TGF-beta signaling. J. Immunother. Cancer *10*, e003750.

12. Schlabach, M.R., Lin, S., Collester, Z.R., Wrocklage, C., Shenker, S., Calnan, C., Xu, T., Gannon, H.S., Williams, L.J., Thompson, F., et al. (2023). Rational design of a SOCS1-edited tumor-infiltrating lymphocyte therapy using CRISPR/Cas9 screens. J. Clin. Investig. *133*, e163096.

13. Chamberlain, C.A., Bennett, E.P., Kverneland, A.H., Svane, I.M., Donia, M., and Met, Ö. (2022). Highly efficient PD-1-targeted CRISPR-Cas9 for tumor-infiltrating lymphocyte-based adoptive T cell therapy. Mol. Ther. Oncolytics *24*, 417–428.

14. Nahmad, A.D., Reuveni, E., Goldschmidt, E., Tenne, T., Liberman, M., Horovitz-Fried, M., Khosravi, R., Kobo, H., Reinstein, E., Madi, A., et al. (2022). Frequent aneuploidy in primary human T cells after CRISPR-Cas9 cleavage. Nat. Biotechnol. *40*, 1807–1813.

15. Weisheit, I., Kroeger, J.A., Malik, R., Klimmt, J., Crusius, D., Dannert, A., Dichgans, M., and Paquet, D. (2020). Detection of Deleterious On-Target Effects after HDR-Mediated CRISPR Editing. Cell Rep. *31*, 107689.

16. Cullot, G., Boutin, J., Toutain, J., Prat, F., Pennamen, P., Rooryck, C., Teichmann, M., Rousseau, E., Lamrissi-Garcia, I., Guyonnet-Duperat, V., et al. (2019). CRISPR-Cas9 genome editing induces megabase-scale chromosomal truncations. Nat. Commun. *10*, 1136.

17. Webber, B.R., Lonetree, C.L., Kluesner, M.G., Johnson, M.J., Pomeroy, E.J., Diers, M.D., Lahr, W.S., Draper, G.M., Slipek, N.J., Smeester, B.A., et al. (2019). Highly efficient multiplex human T cell engineering without double-strand breaks using Cas9 base editors. Nat. Commun. *10*, 5222.

18. Gaudelli, N.M., Komor, A.C., Rees, H.A., Packer, M.S., Badran, A.H., Bryson, D.I., and Liu, D.R. (2017). Programmable base editing of A*T to G*C in genomic DNA without DNA cleavage. Nature *551*, 464–471.

19. Fiumara, M., Ferrari, S., Omer-Javed, A., Beretta, S., Albano, L., Canarutto, D., Varesi, A., Gaddoni, C., Brombin, C., Cugnata, F., et al. (2024). Genotoxic effects of base and prime editing in human hematopoietic stem cells. Nat. Biotechnol. *42*, 877–891.

20. Albarran Fernandez, V., Ballestin Martinez, P., Stoltenborg Granhoj, J., Borch, T.H., Donia, M., and Marie Svane, I. (2024). Biomarkers for response to TIL therapy: a comprehensive review. J. Immunother. Cancer *12*.

21. Jimenez-Reinoso, A., Nehme-Alvarez, D., Dominguez-Alonso, C., and Alvarez-Vallina, L. (2020). Synthetic TILs: Engineered Tumor-Infiltrating Lymphocytes With Improved Therapeutic Potential. Front. Oncol. *10*, 593848.

22. Stadtmauer, E.A., Fraietta, J.A., Davis, M.M., Cohen, A.D., Weber, K.L., Lancaster, E., Mangan, P.A., Kulikovskaya, I., Gupta, M., Chen, F., et al. (2020). CRISPR-engineered T cells in patients with refractory cancer. Science *367*, eaba7365.

23. Brunet, E., and Jasin, M. (2018). Induction of Chromosomal Translocations with CRISPR-Cas9 and Other Nucleases: Understanding the Repair Mechanisms That Give Rise to Translocations. Adv. Exp. Med. Biol. *1044*, 15–25.

24. Yin, J., Lu, R., Xin, C., Wang, Y., Ling, X., Li, D., Zhang, W., Liu, M., Xie, W., Kong, L., et al. (2022). Cas9 exo-endonuclease eliminates chromosomal translocations during genome editing. Nat. Commun. *13*, 1204.

25. Wu, J., Zou, Z., Liu, Y., Liu, X., Zhangding, Z., Xu, M., and Hu, J. (2022). CRISPR/Cas9-induced structural variations expand in T lymphocytes in vivo. Nucleic Acids Res. *50*, 11128–11137.

26. Chauvin, J.M., and Zarour, H.M. (2020). TIGIT in cancer immunotherapy. J. Immunother. Cancer *8*, e000957.

27. Lak, S., Janelle, V., Djedid, A., Boudreau, G., Brasey, A., Lisi, V., Smaani, A., Carli, C., Busque, L., Lavallée, V.P., and Delisle, J.S. (2022). Combined PD-L1 and TIM3 blockade improves expansion of fit human CD8(+) antigen-specific T cells for adoptive immunotherapy. Mol. Ther. Methods Clin. Dev. *27*, 230–245.

28. Gossink, E.M., Coffer, P.J., Cutilli, A., and Lindemans, C.A. (2025). Immunomodulation by galectin-9: Distinct role in T cell populations, current therapeutic avenues and future potential. Cell. Immunol. *407*, 104890.

29. Wolf, Y., Anderson, A.C., and Kuchroo, V.K. (2020). TIM3 comes of age as an inhibitory receptor. Nat. Rev. Immunol. *20*, 173–185.

30. Schatton, T., Itoh, Y., Martins, C., Rasbach, E., Singh, P., Silva, M., Mucciarone, K., Heppt, M.V., Geddes-Sweeney, J., Stewart, K., et al. (2022). Inhibition of Melanoma Cell-Intrinsic Tim-3 Stimulates MAPK-Dependent Tumorigenesis. Cancer Res. *82*, 3774–3784.

31. Gannon, P.O., Harari, A., Auger, A., Murgues, C., Zangiacomi, V., Rubin, O., Ellefsen Lavoie, K., Guillemot, L., Navarro Rodrigo, B., Nguyen-Ngoc, T., et al. (2020). Development of an optimized closed and semi-automatic protocol for Good Manufacturing Practice manufacturing of tumor-infiltrating lymphocytes in a hospital environment. Cytotherapy *22*, 780–791.

32. Bobisse, S., Genolet, R., Roberti, A., Tanyi, J.L., Racle, J., Stevenson, B.J., Iseli, C., Michel, A., Le Bitoux, M.A., Guillaume, P., et al. (2018). Sensitive and frequent identification of high avidity neo-epitope specific CD8 (+) T cells in immunotherapy-naive ovarian cancer. Nat. Commun. *9*, 1092.

33. Kluesner, M.G., Nedveck, D.A., Lahr, W.S., Garbe, J.R., Abrahante, J.E., Webber, B.R., and Moriarity, B.S. (2018). EditR: A Method to Quantify Base Editing from Sanger Sequencing. CRISPR J. *1*, 239–250.

OMTON, Volume *33*

Supplemental information

Highly efficient gene knockout in tumor-infiltrating lymphocytes by adenine base editing

Morteza Hafezi, Raphael Genolet, Leila Hadadi, Bovannak Stewen Chap, Sara Bobisse, Greta Maria Paola Giordano Attianese, Hanan El Jorfi, Daniela Cropp, Laetitia Pericou, Marion Arnaud, Kirsten Scholten, Typhanie Maurouard, Denarda Dangaj Laniti, Alexandre Harari, Bernhard Gentner, Melita Irving, and George Coukos

Tabel S1: ABE matched guide RNA sequences.

Target genes	Guide RNA sequence (no PAM)
TIM3 guide RNA1	5'-CTTACTGTTAGATTTATATC-3'
TIM3 guide RNA2	5'-GTTACCTGGGCCATGTCCCC-3'
TIGIT guide RNA1	5'-CAGGAATACCTGAGCTTTCT-3'
TIGIT guide RNA2	5'-CAGGCCTTACCTGAGGCGAG-3'

Table S2: Primers used for sanger sequencing and NGS analysis.

Nextera adapter-TIM3 forward	5'-TCGTCGGCAGCGTCAGATGTGTATAAGAGACAGAAGTTGATTCCTGGGTGTTCAG-3'
Nextera adapter-TIM3 reverse	5'-GTCTCGTGGGCTCGGAGATGTGTATAAGAGACAGGATTTCCCCTCCAAGTTGAGTA-3'
Nextera adapter-TIGIT forward	5'-TCGTCGGCAGCGTCAGATGTGTATAAGAGACAGCTACCCTGATGGGACGTACACT-3'
Nextera adapter-TIGIT reverse	5'-GTCTCGTGGGCTCGGAGATGTGTATAAGAGACAGCACCTGGAAATGCTTTTAGACC-3'
Nextera adapter forward	5'-TCGTCGGCAGCGTCAGATGTGTATAAGAGACAG-3'
Nextera adapter reverse	5'-GTCTCGTGGGCTCGGAGATGTGTATAAGAGACAG-3'

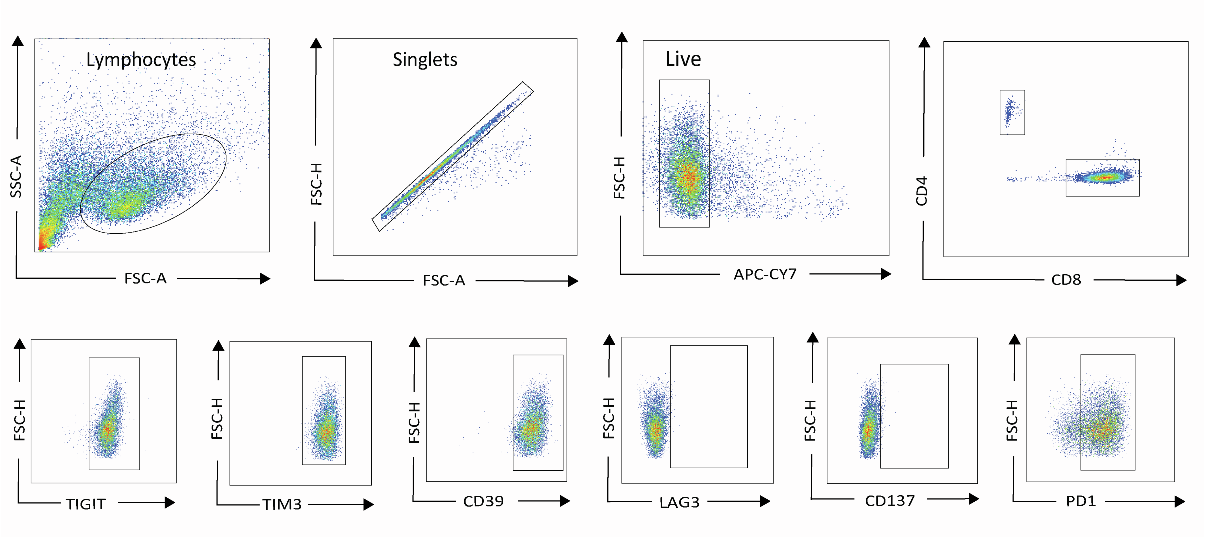

Figure S1: Gating strategy for immunophenotyping of TILs. Exemplary plot for exhaustion and activation markers in live CD4$^+$ and CD8$^+$ TILs. Doublet cells were excluded from the gated TIL population using FSC-A/FSC-H parameters.

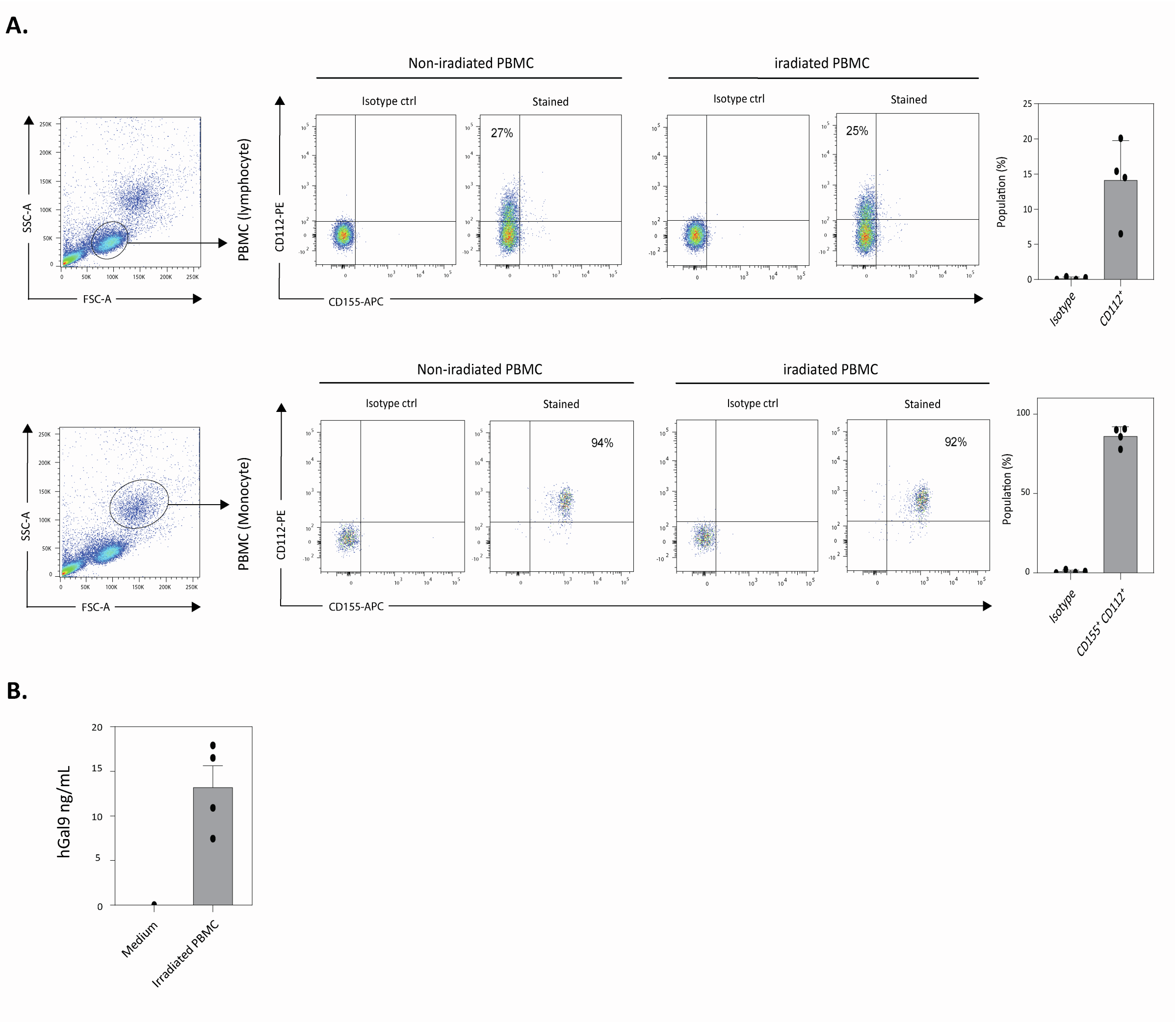

Figure S2: Expression of TIGIT- and TIM3-specific ligands in irradiated feeder cells. (A) Flow cytometry plots demonstrate the expression of TIGIT specific ligands CD112 and CD155 in non-irradiated and irradiated PBMC (**top:** lymphocyte; **bottom:** monocyte). Bar plots represent pooled data from n=4 biological replicates. Isotype control-stained TILs were used as a negative-control for the experiment. Bar plot represents pooled data from n=4 different melanoma-specific TILs (**top:** lymphocyte; **bottom:** monocyte). Lymphocytic and monocytic populations were defined based on their FSC-A and SSC-A parameters. Isotype-stained TILs were used as controls. **(B)** Bar plots represent soluble hGAL9 of irradiated PBMC at day 4 post activation (REP medium). Data pooled data from n=4 biological replicates. REP medium alone was used as negative control.

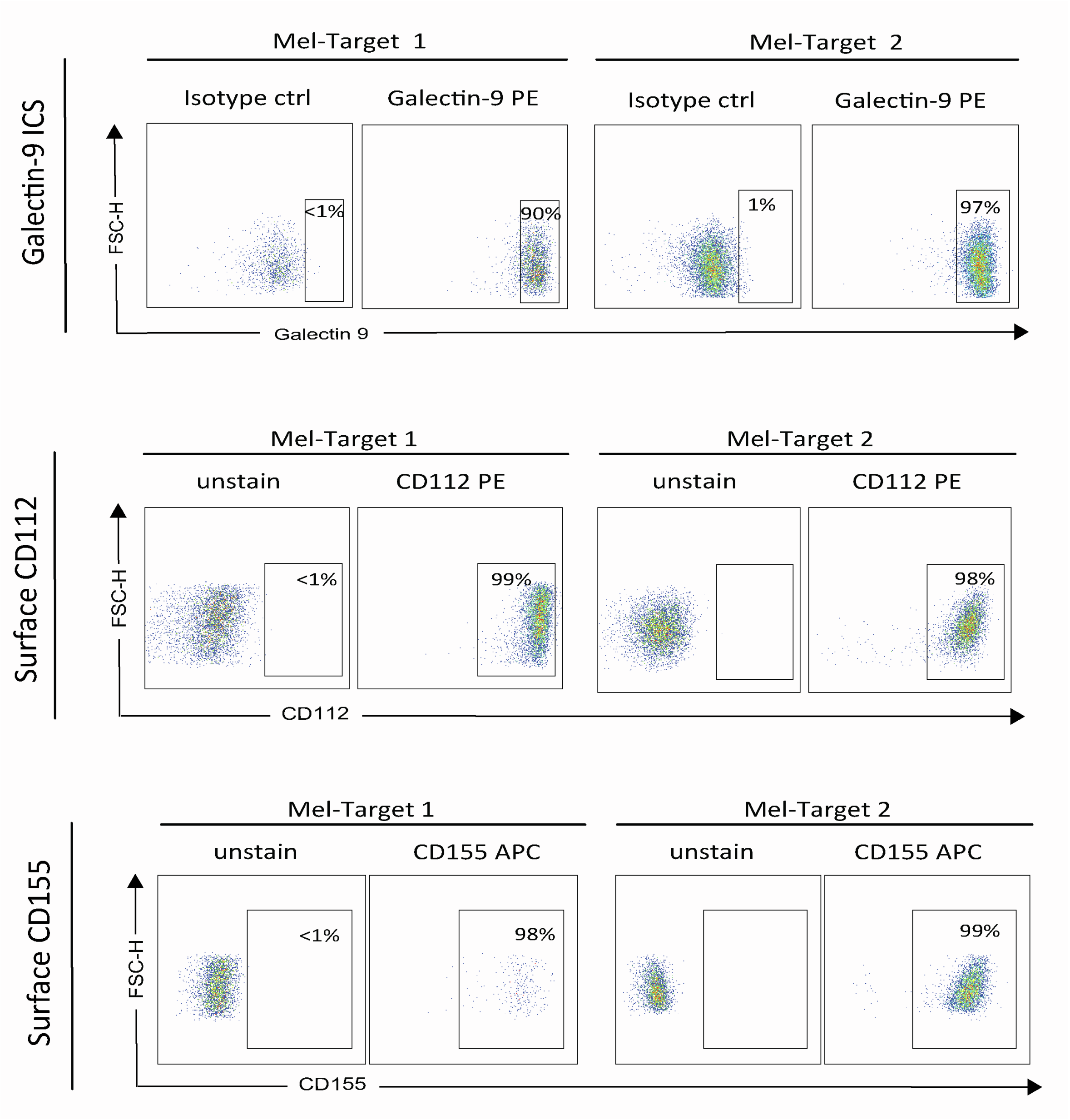

Figure S3: Expression of TIM-3- and TIGIT-specific ligands in patient-derived melanoma cell lines. Exemplary dot plots show the expression of **top**: intracellular staining (ICS) of Galectin-9, **middle**: surface expression of CD112, and **bottom**: surface expression of CD155 in melanoma tumor cell lines.

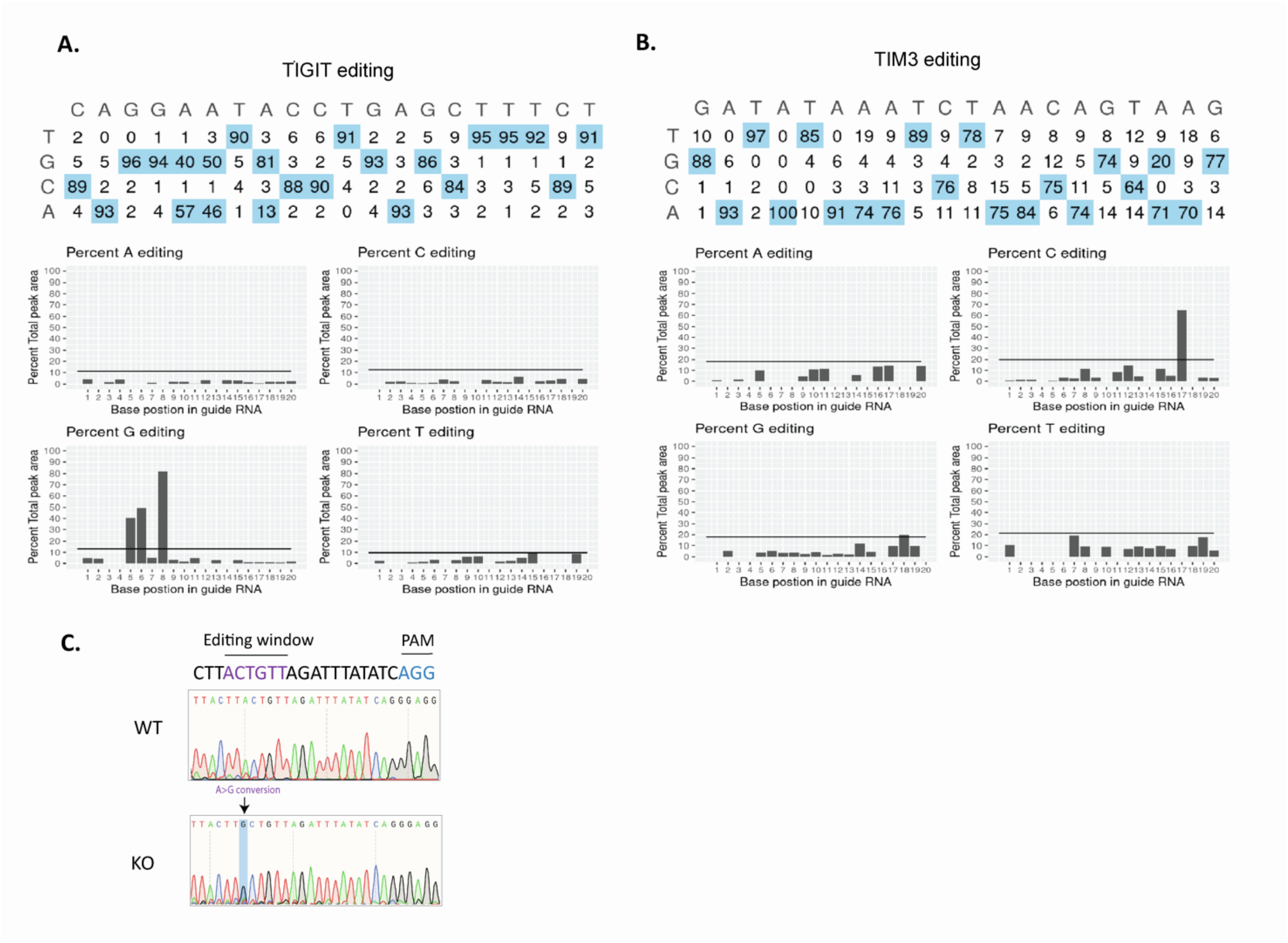

Figure S4: Validation of targeted mutations in ABE-TILs. (**A, B**). **Top:** editing table plot demonstrates the proportion of base conversions within the editing window. **Bottom:** editing bar plots demonstrate the percent of As (antisense Ts) are converted to Gs (antisense Cs). All graphs and plots were generated by analyzing a sanger sequencing file using EditR (n=1). (**C**) Chromatograms obtained by Sanger sequencing demonstrate the point mutation introduced by ABE in exemplary TIM3 edited TILs.

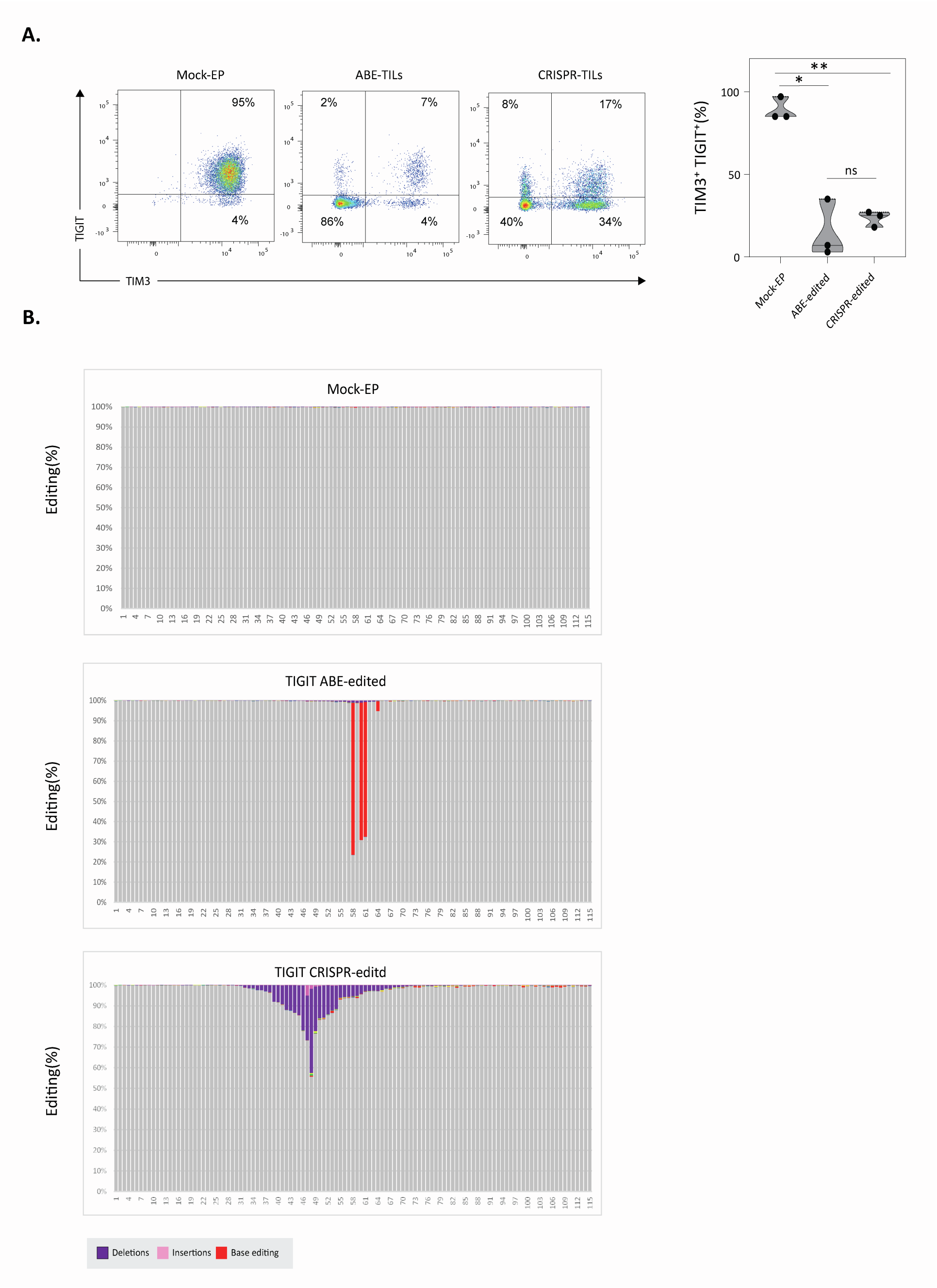

Figure S5: Indel characterization post ABE and CRISPR-CAS9 editing of TILs. (A) Left: Flow cytometry plots demonstrating TIGIT and TIM3 KO in TILs by ABE and CRISPR-Cas9 editing. **Right:** Plot demonstrating % dual KO of TIGIT and TIM3 in ABE versus CRISPR-Cas9 edited TILs (n=3 different melanoma specific TIL samples). **(B)** Exemplary plots showing indel events as evaluated by deep sequencing post ABE (**middle**) or CRISPR-Cas9 (**bottom**) editing in the TIGIT locus. Plot is exemplary of n=3 different melanoma-specific TILs (purple: deletion events; pink: insertion events; red: base editing events). Statistical analysis was performed by one-way ANOVA with Tukey's multiple comparison test. Statistical significance indicated as *P < 0.05, **P < 0.01. ns, not significant.

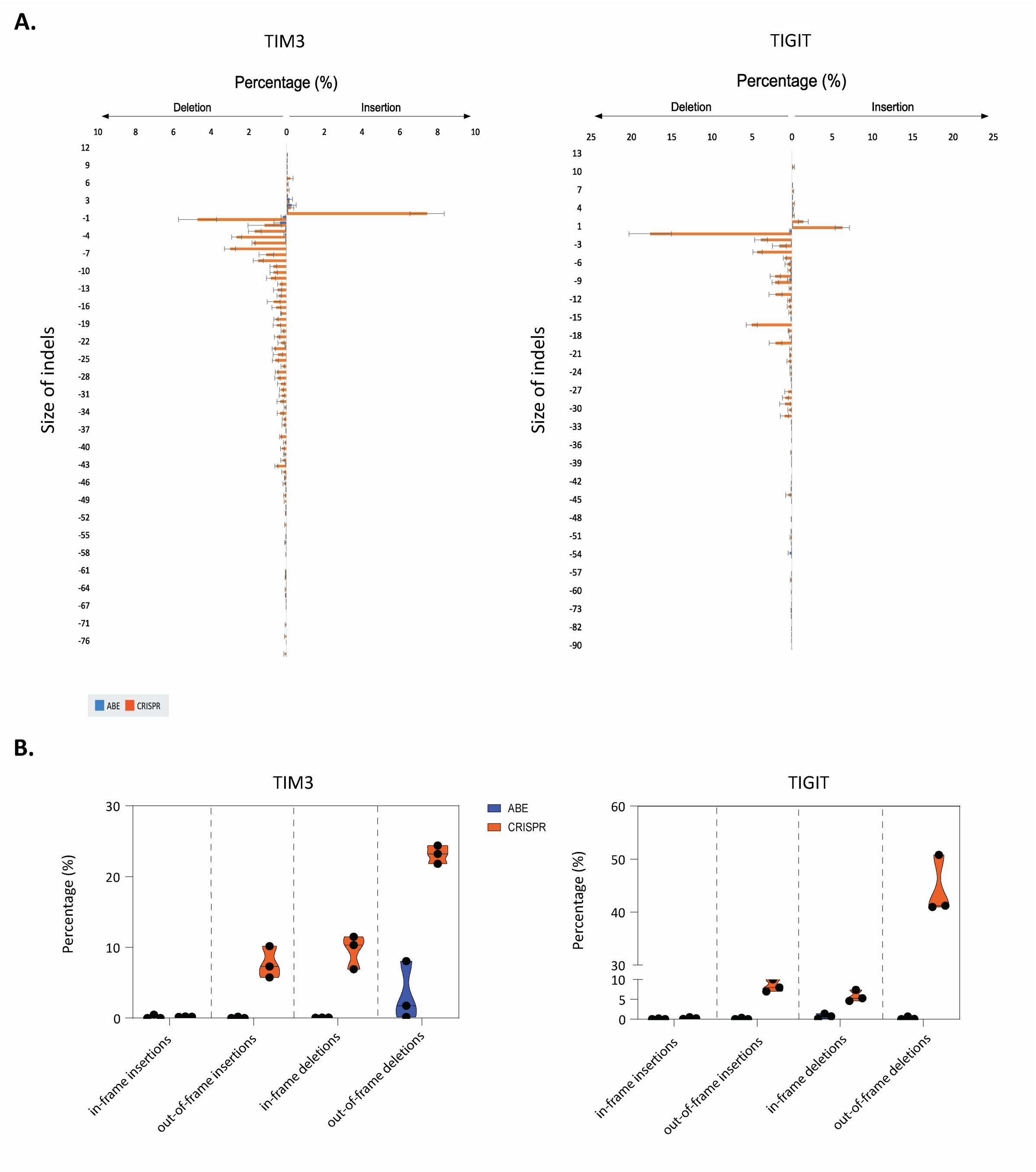

Figure S6: Analysis of ABE versus CRISPR-Cas9 edited TILs. (A) Plot demonstrating the frequency and size of indels for both TIM3 (left) and TIGIT (right) target locus in both CRISPR and ABE edited TILs analyzed by NGS (n=3 biological replicates). **(B)** Violin plots showing percentage of transcripts with in-frame insertion, out-frame insertion, in-frame deletion and out-frame deletion in in both CRISPR and ABE edited TILs. For (B), all values are subtracted from background observed in mock-electroporated (i.e., WT genome) TILs.

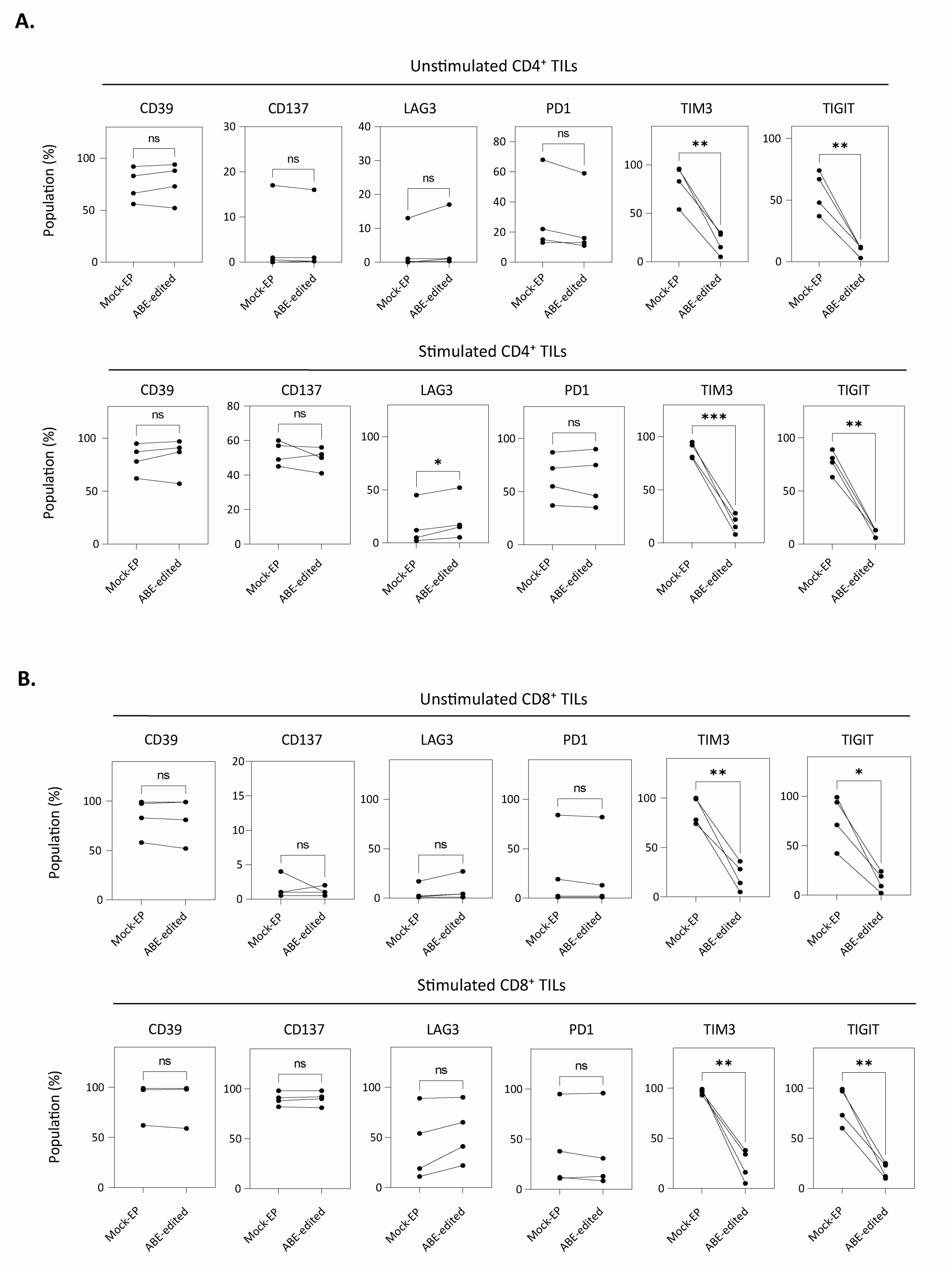

Figure S7: Phenotype of adenine based edited versus non-edited TILs. (A-B) Bar plots demonstrating activation/exhaustion markers in resting (top) and stimulated (bottom) **(A)** $CD4^+$ and **(B)** $CD8^+$ subset of Ova- and Mel-TILs. For Statistical analysis paired t test was performed. Statistical significance indicated as *P < 0.05, **P < 0.01 and ***P < 0.001. ns; not significant.

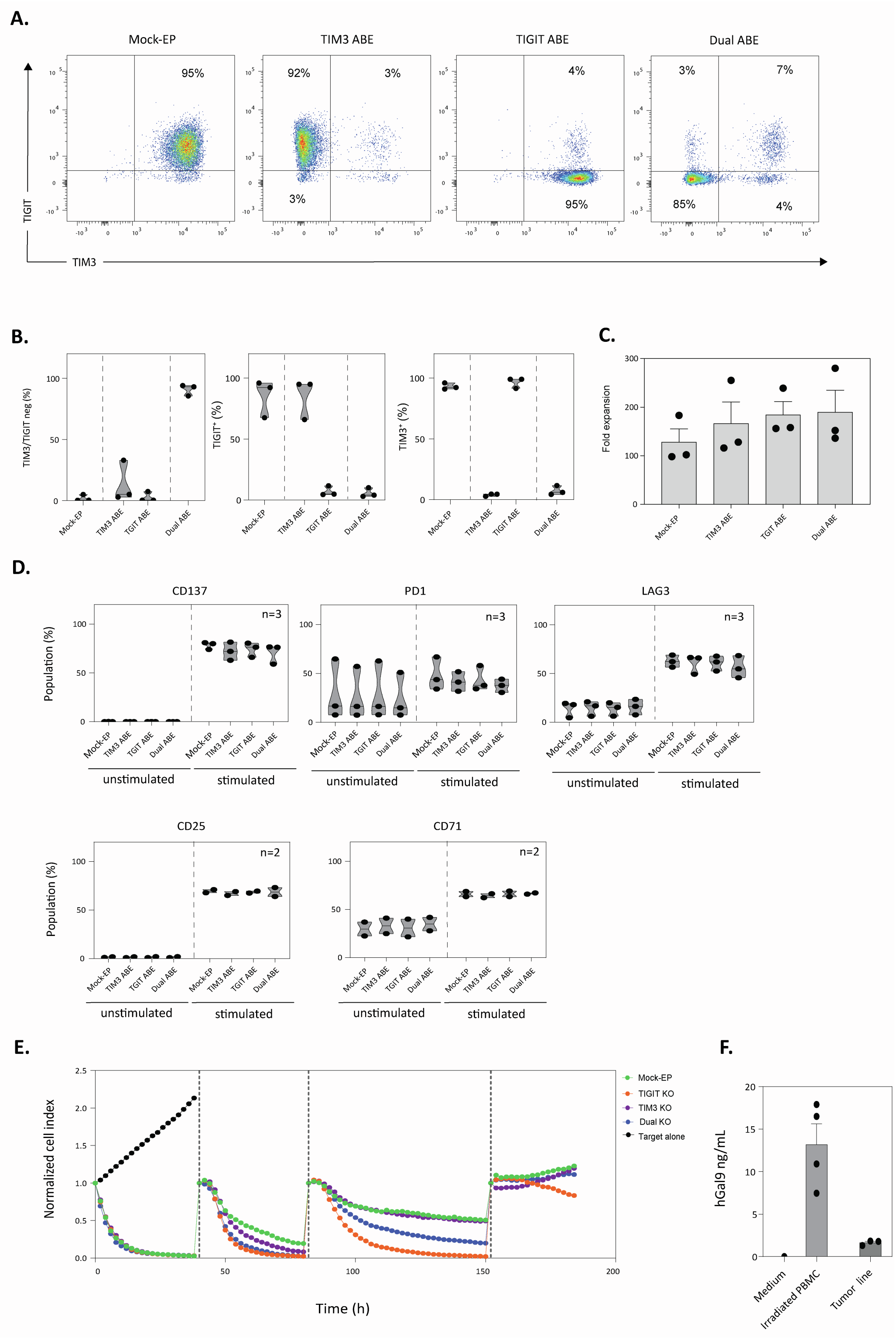

Figure S8: Comparison of single and dual TIM3 and TIGIT ABE-edited TILs (A) Exemplary dot plot showing single and dual KO efficiency in ABE-edited TILs. **(B)** Violin plots demonstrating editing efficiency (n=3 biological replicates). **Left:** percentage of double negative TIM3/TIGIT TILs, **middle:** percentage of TIGIT⁺ TILs, **right:** percentage of TIM3⁺ TILs. **(C)** Fold-expansion calculated at the end of REP phase for all edited and non-edited

TILs (n=3 biological replicates). **(D)** Immunophenotyping of single and dual ABE-edited TILs. Violin plots demonstrate activation/exhaustion markers in unstimulated and stimulated TILs. The number of biological replicates is shown in each violin plot. **(E)** Exemplary plot showing serial cytolytic capacity of TILs determined by a real-time killing assay. The plot is exemplary of 3 independent experiments. Edited and non-edited TILs were co-cultured at an effector-to-target ratio of 2:1 with autologous melanoma target cells. T cell-mediated cytotoxicity was determined by measuring GFP-positive tumor cells at 2-hour intervals for approximately 90 hours post T cell addition using the Incucyte S3 system. **(F)** Bar plots represent soluble hGAL9 levels for both irradiated PBMCs at day 4 post-activation and the patient-derived melanoma line. Data pooled data from n=4 biological replicates for PBMC and n=3 technical replicates of patient-derived tumor line. REP medium alone is used as a negative control.

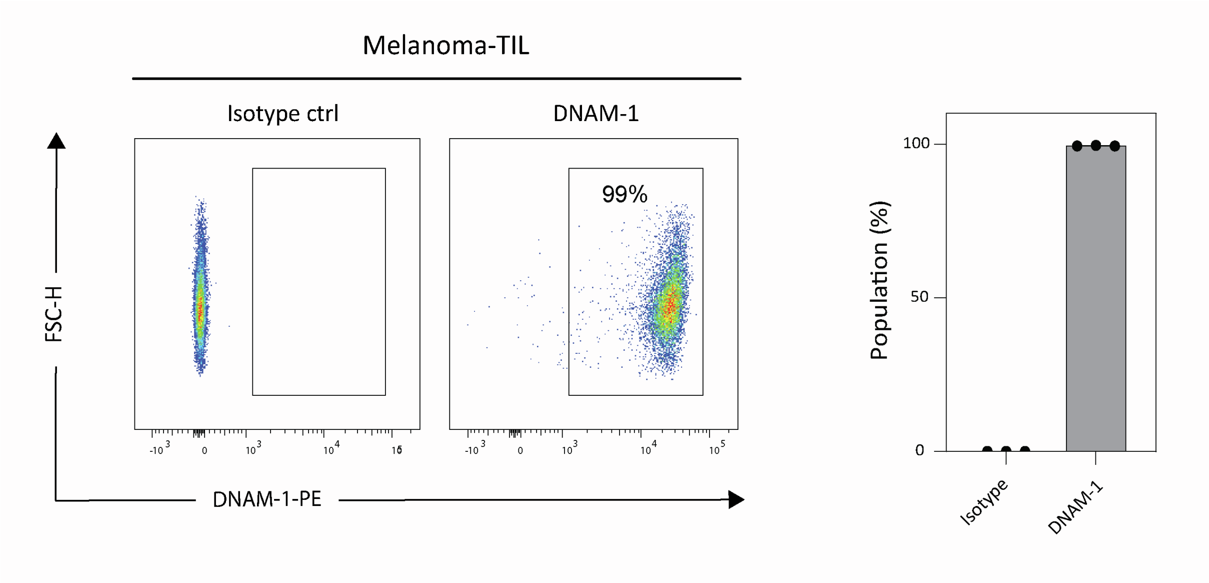

Figure S9: Evaluation of DNAM-1 expression on REP-TILs. Exemplary flow cytometry plots show the expression of DNAM-1 melanoma TILs. Bar plots represent pooled data from n=3 different melanoma-specific TILs. Isotype control-stained (Isotype ctrl) TILs were used as negative-control for the experiment.

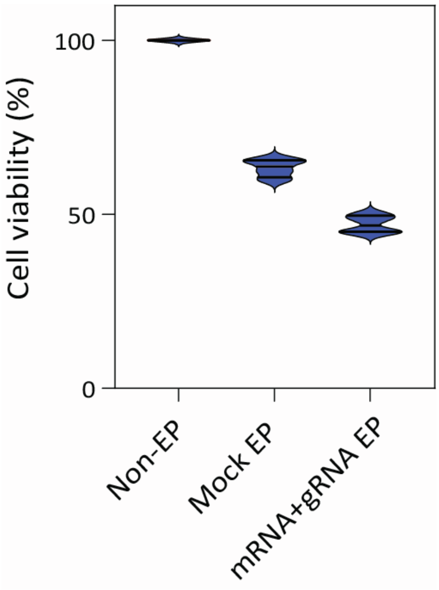

Figure S10: Viability of TILs post-electroporation. Bar plots represent pooled data from 4 different TIL samples. Electroporated (EP) and non-EP TILs were counted after EP using Trypan blue. Non-EP TIL counts were set to 100%.

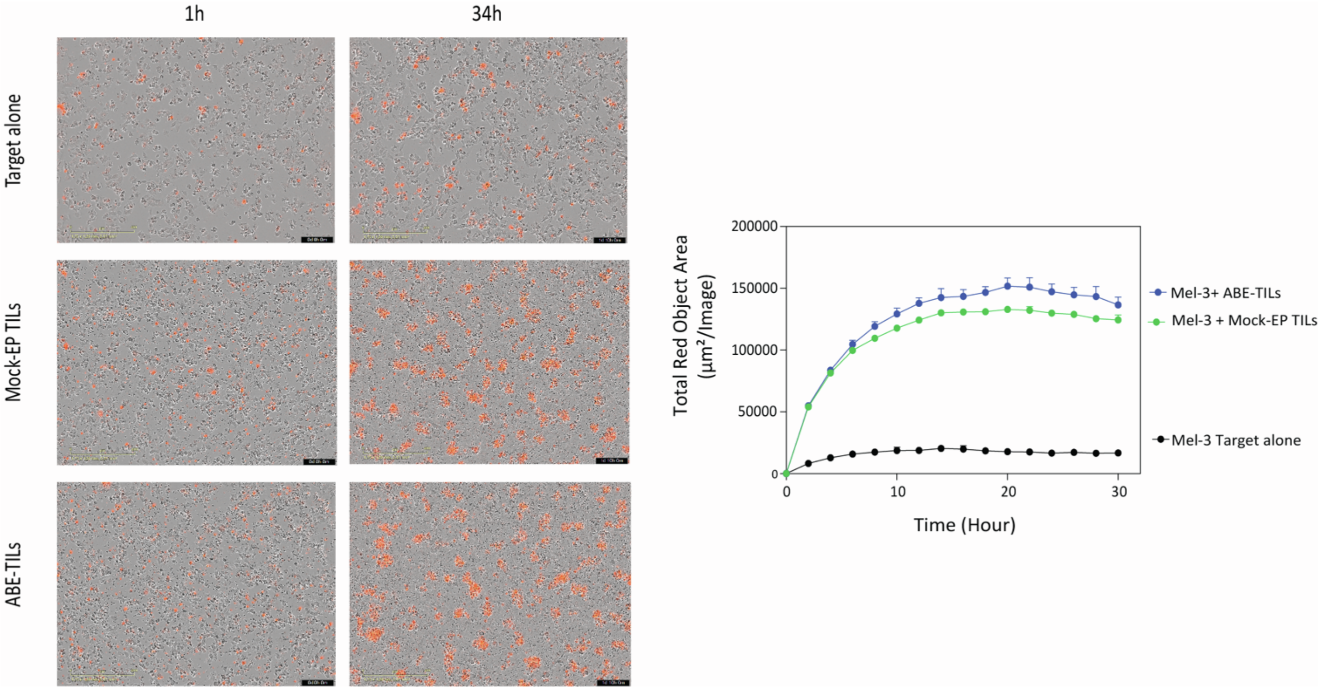

Figure S11: Dual TIM3 and TIGIT ABE-TILs retain unaltered killing capacity in the initial tumor cell challenge. Left: Exemplary images show the killing efficiency of Mock and ABE-EP TILs after 35 hours co-culture. ABE- and Mock-EP TILs were co-cultured at an effector: target ratio of 1:1 with autologous melanoma target cells (Mel-3 target cell line). **Right:** Plot illustrates T cell-mediated cytotoxicity evaluated using the IncuCyte S3 system by measuring Cytox red positive cells at 2-hour intervals for approximately 35 hours post co-culture initiation. Melanoma target cells alone were used as a negative control.

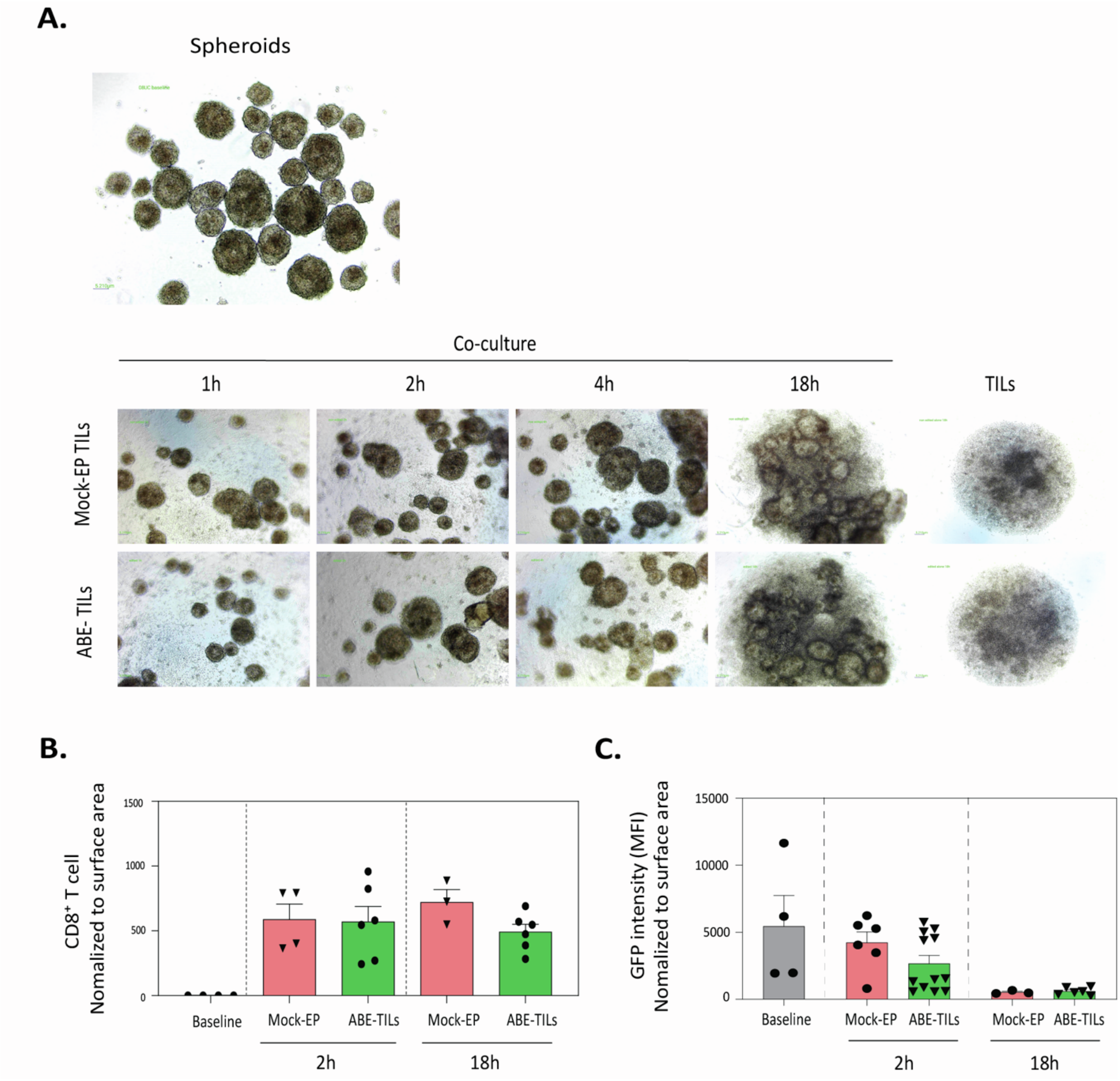

Figure S12: Characterization of melanoma spheroid-TIL co-cultures. (A) Exemplary brightfield microscopy images of spheroids alone and co-cultured with Mock-EP and ABE-TILs at different time-points. Images were acquired using the 5x objective. **(B)** evaluation of T cell infiltration in tumor spheroids at 2 and 18 hours post-co-culture. **(C)** GFP fluorescence intensity within the region of interest (ROI) in the spheroid at baseline, and after 2 and 18 hours co-culture.

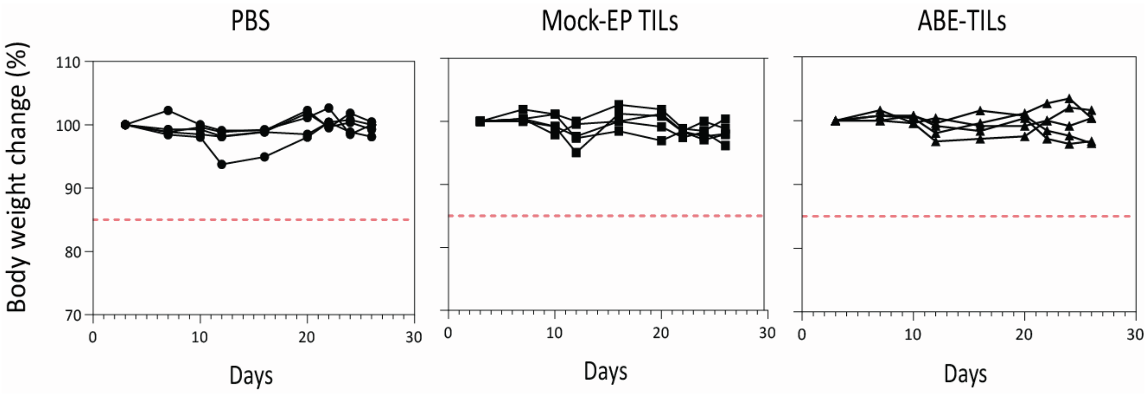

Figure S13: Weight measurements of NSG mice following TIL administration. Graphs illustrate body weight changes for mice over time following control PBS injections versus Mock-EP + IL-2 or ABE-TIL + IL-2 treatments. Each line represents the percentage of body weight changes in a single mouse. Initial body weight was set as 100% and the red dashed line marks 15% weight loss at which point mice would be sacrificed according to regulations.
